# Surveillance for Violent Deaths — National Violent Death Reporting System, 18 States, 2014

**DOI:** 10.15585/mmwr.ss6702a1

**Published:** 2018-02-02

**Authors:** Katherine A. Fowler, Shane P.D. Jack, Bridget H. Lyons, Carter J. Betz, Emiko Petrosky

**Affiliations:** 1 Division of Violence Prevention, National Center for Injury Prevention and Control, CDC, Atlanta, Georgia

## Abstract

**Problem/Condition:**

In 2014, approximately 59,000 persons died in the United States as a result of violence-related injuries. This report summarizes data from CDC’s National Violent Death Reporting System (NVDRS) regarding violent deaths from 18 U.S. states for 2014. Results are reported by sex, age group, race/ethnicity, marital status, location of injury, method of injury, circumstances of injury, and other selected characteristics.

**Reporting Period Covered:**

2014.

**Description of System:**

NVDRS collects data from participating states regarding violent deaths. Data are obtained from death certificates, coroner/medical examiner reports, law enforcement reports, and secondary sources (e.g., child fatality review team data, supplemental homicide reports, hospital data, and crime laboratory data). This report includes data from 18 states that collected statewide data for 2014 (Alaska, Colorado, Georgia, Kentucky, Maryland, Massachusetts, Michigan, New Jersey, New Mexico, North Carolina, Ohio, Oklahoma, Oregon, Rhode Island, South Carolina, Utah, Virginia, and Wisconsin). NVDRS collates documents for each death and links deaths that are related (e.g., multiple homicides, a homicide followed by a suicide, or multiple suicides) into a single incident.

**Results:**

For 2014, a total of 22,098 fatal incidents involving 22,618 deaths were captured by NVDRS in the 18 states included in this report. The majority of deaths were suicides (65.6%), followed by homicides (22.5%), deaths of undetermined intent (10.0%), deaths involving legal intervention (1.3%) (i.e., deaths caused by law enforcement and other persons with legal authority to use deadly force, excluding legal executions), and unintentional firearm deaths (<1%). The term “legal intervention” is a classification incorporated into the *International Classification of Diseases, Tenth Revision (ICD-10)* and does not denote the lawfulness or legality of the circumstances surrounding a death caused by law enforcement. Suicides occurred at higher rates among males, non-Hispanic American Indian/Alaska Natives (AI/AN), non-Hispanic whites, persons aged 45–54 years, and males aged ≥75 years. Suicides were preceded primarily by a mental health, intimate partner, substance abuse, or physical health problem or a crisis during the previous or upcoming 2 weeks. Homicide rates were higher among males and persons aged <1 year and 15–44 years; rates were highest among non-Hispanic black and AI/AN males. Homicides primarily were precipitated by arguments and interpersonal conflicts, occurrence in conjunction with another crime, or related to intimate partner violence (particularly for females). When the relationship between a homicide victim and a suspected perpetrator was known, it was most often either an acquaintance/friend or an intimate partner. Legal intervention death rates were highest among males and persons aged 20–44 years; rates were highest among non-Hispanic black males and Hispanic males. Precipitating factors for the majority of legal intervention deaths were alleged criminal activity in progress, the victim reportedly using a weapon in the incident, a mental health or substance abuse problem, an argument or conflict, or a recent crisis. Deaths of undetermined intent occurred more frequently among males, particularly non-Hispanic black and AI/AN males, and persons aged 30–54 years. Substance abuse, mental health problems, physical health problems, and a recent crisis were the most common circumstances preceding deaths of undetermined intent. Unintentional firearm deaths were more frequent among males, non-Hispanic whites, and persons aged 10–24 years; these deaths most often occurred while the shooter was playing with a firearm and were most often precipitated by a person unintentionally pulling the trigger or mistakenly thinking the firearm was unloaded.

**Interpretation:**

This report provides a detailed summary of data from NVDRS for 2014. The results indicate that violent deaths resulting from self-inflicted or interpersonal violence disproportionately affected persons aged <65 years, males, and certain minority populations. The primary precipitating factors for homicides and suicides were intimate partner problems, interpersonal conflicts, mental health and substance abuse problems, and recent crises.

**Public Health Action:**

NVDRS data are used to monitor the occurrence of violence-related fatal injuries and assist public health authorities in the development, implementation, and evaluation of programs and policies to reduce and prevent violent deaths. For example, North Carolina VDRS data were used to improve case ascertainment of pregnancy-associated suicides, Wisconsin VDRS data were used to develop the statewide suicide prevention strategy, and Colorado VDRS data were used to develop programs and prevention strategies for suicide among veterans. The continued development and expansion of NVDRS to include all U.S. states, territories, and the District of Columbia are essential to public health efforts to reduce the impact of violence.

## Introduction

In 2014, approximately 59,000 persons died in the United States as a result of violence-related injuries (*1*). Suicide was the 10th leading cause of death overall in the United States and disproportionately affected young and middle-aged populations. It was among the top two leading causes of death for persons aged 10–34 years and among the top four for persons aged 35–54 years. Non-Hispanic American Indian/Alaska Native and non-Hispanic white males were disproportionally affected by suicide.

Homicide was the 17th leading cause of death overall in the United States and disproportionately affected young persons (*1*). It was the third leading cause of death for persons aged 15–34 years, the fourth leading cause of death for children aged 1–9 years, and the fifth leading cause of death for persons aged 10–14 years. Homicide disproportionately affected young black males; it was the leading cause of death among non-Hispanic black males aged 15–34 years.

Public health authorities require accurate, timely, and comprehensive surveillance data to better understand and ultimately prevent the occurrence of violent deaths in the United States (*2*). In 2000, in response to an Institute of Medicine* report noting the need for a national fatal intentional injury surveillance system (*3*), CDC began planning to implement the National Violent Death Reporting System (NVDRS) (*2*). The goals of NVDRS are to:

• collect and analyze timely, high-quality data for monitoring the magnitude and characteristics of violent deaths at national, state, and local levels;

• ensure data are disseminated routinely and expeditiously to public health officials, law enforcement officials, policymakers, and the public;

• ensure data are used to develop, implement, and evaluate programs and strategies that are intended to reduce and prevent violent deaths and injuries at national, state, and local levels; and

• build and strengthen partnerships among organizations and communities at national, state, and local levels to ensure that data are collected and used to reduce and prevent violent deaths and injuries.

NVDRS is a state-based active surveillance system that collects data on the characteristics and circumstances associated with all violence-related deaths in participating states. Data on deaths include homicides, suicides, legal intervention deaths (i.e., deaths caused by law enforcement acting in the line of duty and other persons with legal authority to use deadly force but excluding legal executions), unintentional firearm deaths, and deaths of undetermined intent.^†^ The term “legal intervention” is a classification incorporated into the *International Classification of Diseases, Tenth Revision (ICD-10)* and does not denote the lawfulness or legality of the circumstances surrounding a death caused by law enforcement. NVDRS data are used to assist the development, implementation, and evaluation of programs and strategies designed to reduce and prevent these deaths at the national, state, and local levels.

Before implementation of NVDRS, single data sources (e.g., death certificates or law enforcement reports) provided only limited information and few circumstances from which to understand patterns of violent deaths. NVDRS fills this surveillance gap by providing more detailed information. It is the first system to 1) provide detailed information on circumstances precipitating violent deaths, 2) link multiple source documents on violent deaths so that each incident can contribute to the study of patterns of violent deaths, and 3) link multiple deaths that are related to one another (e.g., multiple homicides, suicide pacts, or homicide followed by the suicide of the suspected perpetrator).

NVDRS began data collection in 2003 with six states (Maryland, Massachusetts, New Jersey, Oregon, South Carolina, and Virginia). Seven states (Alaska, Colorado, Georgia, North Carolina, Oklahoma, Rhode Island, and Wisconsin) joined in 2004; four (California, Kentucky, New Mexico, and Utah) joined in 2005; and two (Ohio and Michigan) joined in 2010 (Figure). CDC provides funding for state participation, and the ultimate goal is for NVDRS to expand to include all 50 states, U.S. territories, and the District of Columbia.^§^

**FIGURE:**
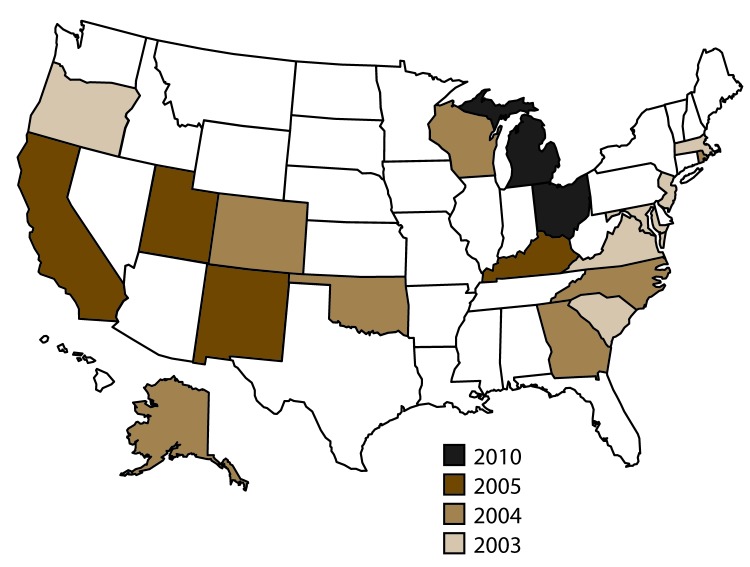
States participating in the National Violent Death Reporting System, by year of initial data collection, United States, 2003–2014

This report summarizes data for 2014 for deaths meeting NVDRS inclusion criteria from the 18 states that collected statewide data in that year (Alaska, Colorado, Georgia, Kentucky, Maryland, Massachusetts, Michigan, New Jersey, New Mexico, North Carolina, Ohio, Oklahoma, Oregon, Rhode Island, South Carolina, Utah, Virginia, and Wisconsin); these states account for approximately 33.4% of the U.S. population (*1*,*4*). The analysis in this report does not include data from California, which concluded its participation in 2009. NVDRS data are updated annually and are publicly available through CDC’s Web-based Injury Statistics Query and Reporting System (WISQARS)^¶^ at https://www.cdc. gov/injury/wisqars/nvdrs.html. Case-level NVDRS data are available to applicants who meet eligibility requirements via access to the NVDRS Restricted Access Database (https://www.cdc.gov/ViolencePrevention/NVDRS/RAD.html).

## Methods

NVDRS compiles information from multiple data sources. The core required data sources are death certificates, coroner/medical examiner reports, and law enforcement reports. Some participating states also collect information from secondary sources (e.g., child fatality review team data, supplemental homicide reports, and crime laboratory data). NVDRS collates documents for each death and links deaths that are related (e.g., multiple homicides, a homicide followed by a suicide, or multiple suicides) into a single incident. The ability to analyze linked data permits comprehensive assessment of violent deaths. This report presents select data for 2014. Additional data from 2014 are available at https://stacks.cdc.gov/view/cdc/50765.

In NVDRS, a violent death is defined as a death resulting from the intentional use of physical force or power, threatened or actual, against oneself, another person, or a group or community (*5*). Information also is collected about unintentional firearm deaths (i.e., a death resulting from a penetrating injury or gunshot wound from a weapon that uses a powder charge to fire a projectile when there was a preponderance of evidence that the shooting was not intentionally directed at the victim) and deaths of undetermined intent (i.e., a death that results from the use of force or power against oneself or another person for which the evidence indicating one manner of death is no more compelling than evidence indicating another). NVDRS cases are coded according to *ICD-10 *guidelines (*6*) or on the basis of the manner of death assigned by the coroner/medical examiner or law enforcement. Cases are included if they are assigned *ICD-10* codes (Box 1) or if the manner of death specified in at least one of the three primary data sources is consistent with NVDRS case definitions.

BOX 1 ***International Classification of Diseases, Tenth Revision* *(ICD-10)* codes used in the National Violent Death Reporting System**

**Table:** 

**Manner of death**	**Death ≤1 year after injury**	**Death >1 year after injury**
Intentional self-harm (suicide)	X60–X84	Y87.0
Assault (homicide)	X85–X99, Y00–Y09	Y87.1
Event of undetermined intent	Y10–Y34	Y87.2, Y89.9
Unintentional exposure to inanimate mechanical forces (firearms)	W32–W34	Y86 determined to be attributable to firearms
Legal intervention (excluding executions, Y35.5)	Y35.0–Y35.4, Y35.6–Y35.7	Y89.0
Terrorism	U01, U03	U02

Variables analyzed in NVDRS include:

• manner of death (i.e., the intent [homicide/legal intervention, suicide, unintentional, undetermined] of the person inflicting a fatal injury);

• mechanism of injury (i.e., the method used to inflict a fatal injury) (Box 2);

BOX 2 **Methods used to inflict injury — National Violent Death Reporting System, 18 states, 2014**

**Table:** 

Firearm: method that uses a powder charge to fire a projectile Hanging/strangulation/suffocation: hanging by the neck, manual strangulation, or plastic bag over the head Poisoning: street drug, alcohol, pharmaceutical, carbon monoxide, gas, rat poison, or insecticide Sharp instrument: knife, razor, machete, or pointed instrument (e.g., chisel or broken glass) Blunt instrument: (e.g., club, bat, rock, or brick) Fall: being pushed or jumping Motor vehicle: (e.g., car, bus, motorcycle, or other transport vehicle) Personal weapons: (e.g., hands, fists, or feet) Drowning: inhalation of liquid in bathtub, lake, or other source of water/liquid Fire/burns: inhalation of smoke or the direct effects of fire or chemical burns Intentional neglect: starvation, lack of adequate supervision, or withholding of health care Other: any method other than those already listed Unknown: method not reported or not known

• toxicology findings (for decedents who were tested);

• circumstances preceding injury (i.e., the events that preceded and were identified by investigators as relevant and therefore might have contributed to the infliction of a fatal injury) (Box 3);

BOX 3 **Circumstances preceding fatal injury, by manner of death — National Violent Death Reporting System, 18 states, 2014**

**Table:** 

**Suicide/Undetermined Intent** Intimate partner problem: decedent was experiencing problems with a current or former intimate partner. Suicide of friend or family: decedent was distraught over, or reacting to, the suicide of a friend or family member. Other death of friend or family: decedent was distraught over, or reacting to, the nonsuicide death of a friend or family member. Physical health problem: decedent was experiencing physical health problems (e.g., a recent cancer diagnosis or chronic pain). Job problem: decedent was either experiencing a problem at work or was having a problem with joblessness. Recent criminal legal problem: decedent was facing criminal legal problems. Noncriminal legal problem: decedent was facing civil legal problems (e.g., a child custody or civil lawsuit). Financial problem: decedent was experiencing problems such as bankruptcy, overwhelming debt, or foreclosure of a home or business. Eviction or loss of home: decedent was experiencing a recent eviction or other loss of housing. School problem: decedent was experiencing a problem such as poor grades, bullying, social exclusion at school, or performance pressures. Traumatic anniversary: the incident occurred on or near the anniversary of a traumatic event in the decedent’s life. Exposure to disaster: decedent was exposed to a disaster (e.g., earthquake or bombing). Left a suicide note: decedent left a note, e-mail message, video, or other communication indicating intent to die by suicide. Disclosed intent to die by suicide: decedent had previously expressed suicidal feelings to another person with time for that person to intervene. History of suicide thoughts or plans: decedent had previously expressed suicidal thoughts or plans. History of suicide attempts: decedent had previously attempted suicide before the fatal incident. **Homicide/Legal Intervention** Jealousy (lovers' triangle): jealousy or distress over an intimate partner's relationship or suspected relationship with another person. Stalking: pattern of unwanted harassing or threatening tactics by either the decedent or suspect. Prostitution: prostitution or related activity that includes prostitutes, pimps, clients, or others involved in such activity. Drug involvement: drug dealing, drug trade, or illegal drug use. Brawl: mutual physical fight involving three or more persons. Mercy killing: decedent wished to die because of terminal or hopeless disease or condition, and documentation indicates that the decedent wanted to be killed. Victim was a bystander: decedent was not the intended target in the incident (e.g., pedestrian walking past a gang fight). Victim was a police officer on duty: decedent was a law enforcement officer killed in the line of duty. Victim was an intervener assisting a crime victim: decedent was attempting to assist a crime victim at the time of the incident (e.g., child attempts to intervene and killed while trying to assist a parent who is being assaulted). Victim used a weapon: decedent used a weapon to attack or defend during the course of the incident. Intimate partner violence-related: incident is related to conflict between current or former intimate partners; includes the death of actual intimate partners and nonintimate partner victims killed to cause pain to an intimate partner (e.g., child or parent). Hate crime: decedent was intentionally selected because of his/her actual or perceived gender, religion, sexual orientation, race/ethnicity, or disability. Mentally ill suspect: suspect’s attack on decedent believed to be the direct result of a mental illness. Drive-by shooting: suspect drove near the decedent and fired a weapon while driving. Walk-by assault: decedent was killed by a targeted attack (e.g., ambush) where the suspect fled on foot. Random violence: decedent was killed by a random act of violence. Gang-related: incident resulted from gang activity or gang rivalry; not used if the decedent was a gang member and the death did not appear to result from gang activity. **All Manners of Death (except Unintentional Firearm)** Current depressed mood: decedent was perceived by self or others to be depressed. Current diagnosed mental health problem: decedent was identified as having a mental health disorder or syndrome listed in the Diagnostic and Statistical Manual, Version IV (DSM-IV), with the exception of alcohol and other substance dependence (these are captured in separate variables). Type of mental health diagnosis: identifies the DSM-IV diagnosis made by a medical or mental health practitioner. Current mental health treatment: decedent was currently receiving mental health treatment as evidenced by a current prescription for a psychotropic medication or visit to a mental health professional in the previous 2 months. History of treatment for mental health problem: decedent was identified as having ever received mental health treatment within the decedent’s lifetime. Alcohol/other substance problem: decedent was perceived by self or others to have a problem with, or to be addicted to, alcohol or other drugs. Other addiction: decedent was perceived by self or others to have an addiction other than alcohol or other substance abuse (e.g., gambling or sexual). Family relationship problem: decedent was experiencing problems with a family member other than an intimate partner. Other relationship problem: decedent was experiencing problems with a friend or associate (other than an intimate partner or family member). History of child abuse/neglect: decedent had history of physical, sexual, or psychological abuse; physical, emotional, or educational neglect; or exposure to a violent environment or inadequate supervision by a caretaker as a child. Caretaker abuse/neglect led to death: decedent was experiencing physical, sexual, or psychological abuse; physical, emotional, or educational neglect; or exposure to a violent environment or inadequate supervision by a caretaker that led to death. Perpetrator of interpersonal violence in previous month: decedent perpetrated interpersonal violence during the previous month. Victim of interpersonal violence in previous month: decedent was the target of interpersonal violence in the past month. Physical fight: a physical fight between two individuals which resulted in the death of the decedent who was either involved in the fight, a bystander, or trying to stop the fight. Argument or conflict: a specific argument or disagreement occurred during the incident. Precipitated by another crime: incident occurred as the result of another serious crime. Nature of crime: identifies the specific type of other crime that occurred during incident (e.g., robbery or drug trafficking). Crime in progress: serious crime was in progress at the time of the incident. Terrorist attack: decedent was injured in a terrorist attack, leading to death. Crisis during previous or upcoming 2 weeks: circumstance that is a current crisis or acute precipitating event that had either occurred in the previous 2 weeks or was impending in the following 2 weeks (e.g., a trial for a criminal offense begins the following week). Other crisis: A crisis related to a death but not captured by any of the circumstances. **Unintentional Firearm Death** ***Context of Injury*** Hunting: death occurred any time after leaving home for a hunting trip and before returning home from a hunting trip. Target shooting: shooter was aiming for a target and unintentionally hit the decedent; can be at a shooting range or an informal backyard setting (e.g., teenagers shooting at signposts on a fence). Loading/unloading gun: gun discharged when the shooter was loading/unloading ammunition. Cleaning gun: shooter pulled trigger or gun discharged while cleaning, repairing, assembling, or disassembling gun. Showing gun to others: showing the gun to another person when the gun discharged or the trigger was pulled. Playing with gun: shooter and one or more others were playing with a gun when it discharged. Celebratory firing: shooter fired gun in celebratory manner (e.g., on New Year’s Eve). Other context of injury: shooting occurred during some context other than those already described. ***Mechanism of Injury*** Unintentionally pulled trigger: shooter unintentionally pulled the trigger (e.g., while grabbing the gun or holding it too tightly). Thought gun safety was engaged: shooter thought the safety was on and gun would not discharge. Thought unloaded/magazine disengaged: shooter thought the gun was unloaded because the magazine was disengaged. Thought gun was unloaded: shooter thought the gun was unloaded for other unspecified reason. Bullet ricochet: bullet ricocheted from its intended target and unintentionally struck the decedent. Gun defect or malfunction: gun had a defect or malfunctioned, as determined by a trained firearm examiner. Gun fired while holstering: gun was being replaced or removed from holster/clothing. Dropped gun: gun discharged when it was dropped or when something was dropped on it. Gun fired while operating safety/lock: shooter unintentionally fired the gun while operating the safety lock. Gun mistaken for toy: gun was mistaken for a toy and was fired without the user understanding the danger. Other mechanism of injury: shooting occurred as the result of a mechanism not already described.

• whether the decedent was a victim (i.e., a person who died as a result of a violence-related injury) or both a suspect and a victim (i.e., a person believed to have inflicted a fatal injury on a victim who then was fatally injured [e.g., the perpetrator of a homicide-suicide]);

• information about any known suspects (i.e., a person believed to have inflicted a fatal injury on a victim);

• incident (i.e., an occurrence in which one or more persons sustained a fatal injury that was linked to a common event or perpetrated by the same suspect during a 24-hour period); and

• type of incident (i.e., a combination of the manner of death and the number of victims in an incident).

NVDRS is an incident-based system, and all decedents associated with a given incident are grouped in one record. Decisions about whether two or more deaths are related and belong to the same incident are made on the basis of the timing of the injuries rather than on the timing of the deaths. Deaths resulting from injuries that occur within 24 hours of each other (i.e., the 24-hour rule) and are clearly linked by source documents (discussed under Manner of Death) would be considered part of the same incident. Examples of an incident include 1) a single isolated violent death, 2) two or more related homicides (including legal intervention deaths) when the fatal injuries were inflicted <24 hours apart, 3) two or more related suicides or deaths of undetermined intent when the fatal injuries were inflicted <24 hours apart, and 4) a homicide followed by a suicide when both fatal injuries were inflicted <24 hours apart (*7*).

Data collected from individual information sources are entered into the NVDRS online data entry system (*2*). In 2013, NVDRS began using a streamlined coding system to facilitate data abstraction efficiency by eliminating the need to enter data into source-specific data entry screens. The streamlined interface allows data from multiple sources to be entered into the same incident record and includes internal validation checks, hover-over features that define selected fields, and other quality control measures. Primacy rules and hierarchal algorithms related to the source documents are now occurring at the state level. Access to the online system is provided to each state by CDC. State project personnel are provided ongoing coding training to help increase data quality. Data are transmitted continually through the web to a CDC-based server. No personally identifiable information is transmitted to CDC.

### Manner of Death

A manner (i.e., intent) of death for each decedent is assigned by a trained abstractor who assimilates information from all source documents. The abstractor-assigned manner of death must agree with at least one core required data source; typically, all source documents are consistent regarding the manner of death. When there is a discrepancy, the abstractor must assign a manner of death on the basis of the preponderance of evidence in the source documents, but such occurrences are rare (*7*). For example, if two sources report a death as a suicide and a third reports it as a death of undetermined intent, the death is coded as a suicide.

NVDRS data are categorized into five abstractor-assigned manners of death: 1) suicide, 2) homicide, 3) unintentional firearm, 4) undetermined intent, and 5) legal intervention.

• **Suicide.** Suicide is a death resulting from the use of force against oneself when a preponderance of evidence indicates that the use of force was intentional. This category also includes the following scenarios: 1) deaths of persons who intended only to injure rather than kill themselves; 2) persons who initially intended to kill themselves, changed their minds, but ultimately died as a result of the act; 3) deaths associated with risk-taking behavior without clear intent to inflict fatal self-injury but associated with high risk for death (e.g., playing Russian roulette); 4) suicide that occurred while under the influence of substances or drugs taken voluntarily; 5) suicide that occurred while under the influence of a mental illness; and 6) suicide involving another person providing only passive assistance to the decedent (e.g., supplying the means or information needed to complete the act). This category does not include deaths caused by chronic or acute substance abuse without the intent to die or deaths attributed to autoerotic behavior (e.g., self-strangulation during sexual activity). Corresponding *ICD-10* codes included in NVDRS are X60–X84 and Y87.0 (Box 1).

• **Homicide.** Homicide is a death resulting from the use of physical force or power, threatened or actual, against another person, group, or community when a preponderance of evidence indicates that the use of force was intentional. Two special scenarios that the National Center for Health Statistics (NCHS) regards as homicides are included in the NVDRS case definition: 1) arson with no specified intent to injure someone and 2) a stabbing with intent unspecified. This category also includes the following scenarios: 1) deaths when the suspect intended only to injure rather than kill the victim, 2) deaths resulting from heart attack induced when the suspect uses force or power against the victim, 3) deaths that occur when a person kills an attacker in self-defense, 4) deaths resulting from a weapon that discharges unintentionally while being used to control or frighten a victim, 5) deaths attributed to child abuse without intent being specified, 6) deaths attributed to an intentional act of neglect by one person against another, 7) death of a child after birth that results from a direct injury due to violence sustained before birth, 8) deaths identified as a justifiable homicide where person committing homicide was not a law enforcement officer, and 9) deaths resulting from an act of terrorism. This category excludes vehicular homicide without intent to injure, unintentional poisoning deaths due to illegal or prescription drug overdose even when the person who provided drugs was charged with homicide, unintentional firearm deaths (a separate category), combat deaths or acts of war, deaths of unborn fetuses, and deaths of children after birth that resulted indirectly from violence sustained by the mother before birth (e.g., death from prematurity following premature labor brought on by violence). Corresponding *ICD-10* codes included in NVDRS are U01–U03, X85–X99, Y00–Y09, and Y87.1 (Box 1).

• **Unintentional firearm.** An unintentional firearm death is a death resulting from a penetrating injury or gunshot wound from a weapon that uses a powder charge to fire a projectile and for which a preponderance of evidence indicates that the shooting was not directed intentionally at the decedent. Examples include a person who dies as a result of celebratory firing that was not intended to frighten, control, or harm anyone; a person who unintentionally shoots himself when using a firearm to frighten, control, or harm another person; a soldier shot during a field exercise but not in a combat situation; a person who received a self-inflicted wound while playing with a firearm; a person who mistakenly believes a gun is unloaded and shoots another person; a child aged <6 years who shoots him- or herself or another person; and a child who dies after birth from an unintentional firearm injury that was sustained in utero. This category excludes firearm injuries caused by unintentionally striking a person with the firearm (e.g., hitting a person on the head with the firearm rather than firing a projectile) and unintentional injuries from nonpowder guns (e.g., BB, pellet, or other compressed air-powered or gas-powered guns). Corresponding *ICD-10* codes included in NVDRS are W32–W34 and Y86 (Box 1).

• **Undetermined intent.** A death of undetermined intent is a death resulting from the use of force or power against oneself or another person for which the evidence indicating one manner of death is no more compelling than evidence indicating another. This category includes coroner/medical examiner rulings (e.g., accident or suicide, undetermined, jumped or fell, or self-inflicted injury) when records give no evidence or opinions in favor of either unintentional or intentional injury. Corresponding *ICD-10* codes in NVDRS are Y10–Y34, Y87.2, and Y89.9 (Box 1).

• **Legal intervention.** A death from legal intervention is a death in which a person is killed or died as a result of a law enforcement officer or other peace officer (i.e., a person with specified legal authority to use deadly force), including military police, while on duty. The term “legal intervention” is a classification from *ICD-10* (Y35.0) and does not denote the lawfulness or legality of the circumstances surrounding the death. Legal intervention deaths include a small subset in which force was applied without clear lethal intent (e.g., during restraint or when applying force with a typically nondeadly weapon such as a Taser [Taser International, Scottsdale, Arizona]) or in which the death occurred while the person was fleeing capture. This category excludes legal executions. Corresponding *ICD-10* codes included in NVDRS are Y35.0–Y35.4, Y35.6, Y35.7, and Y89.0 (Box 1).

### Variables Analyzed

NVDRS collects approximately 600 unique variables for each death. The number of variables recorded for each incident depends on the content and completeness of the source documents. Variables include manner of death; demographic information; *ICD-10* cause of death codes and text descriptors; location, date, and time of injury and death; toxicology results; bodily injuries; precipitating circumstances; victim-suspect relationship; and method of injury (Boxes 1–3).

### Circumstances Preceding Death

The circumstances preceding death are defined as the precipitating events that contributed to the infliction of a fatal injury (Box 3). The circumstances are reported on the basis of the content of the coroner/medical examiner and law enforcement investigative reports. Some circumstances are coded to a specific manner of death (e.g., suicide or death of undetermined intent); other circumstances are coded across all manners of death. The data abstractor selects from a list of potential circumstances and is required to code all known circumstances that relate to each incident. If circumstances are not known (e.g., for a body found in the woods with no other details reported), the data abstractor leaves the “circumstances known” variable blank; these deaths are excluded from the denominator for circumstance values. If either the coroner/medical examiner record or the law enforcement report indicates the presence of a circumstance, the abstractor endorses the circumstance (e.g., if the law enforcement report indicated that a decedent had disclosed an intent to die by suicide, then suicidal intent is endorsed).

### Coding Training and Quality Control

Ongoing coding support for data abstractors is provided through an e-mail help desk, monthly conference calls with all states, and regular conference calls with individual states. States also can conduct additional abstractor training workshops and activities at their own discretion. An NVDRS coding manual (*7*) with CDC-issued standard guidance on coding criteria and examples for each data element is provided. Software features to enhance coding reliability include automated validation rules and a hover-over feature containing variable-specific information.

States are asked to perform annual blind reabstractions of a subset of cases using multiple abstractors to identify inconsistencies. CDC also conducts a quality control analysis in which multiple variables are reviewed for their appropriateness, with special focus on abstractor-assigned variables (e.g., method and manner of death). If CDC finds inconsistencies, the state is notified and asked for a response or correction.

### Time Frame

States are required to report all deaths within 6 months of the end of each calendar year for the preceding January–December. States then have an additional 12 months to complete each incident record. Although states typically meet these requirements, additional details sometimes arrive after a deadline has passed. New incidents also might be identified after the deadline (e.g., a death certificate is revised, new evidence is obtained that changes a manner of death, or an *ICD-10* miscoding is corrected to meet NVDRS inclusion criteria). These additional data are incorporated into NVDRS. Analysis files are updated in real time in the online system. CDC estimates that case counts might increase 1.0%–2.0% after the 18-month data collection period.

### Fatal Injuries in 2014

This report provides preliminary data concerning fatal injuries meeting the NVDRS case definition for violent deaths in 2014 that were received by CDC as of August 9, 2016 for 18 participating states. Participating states used vital statistics death certificate files or coroner/medical examiner reports to identify violent deaths meeting NVDRS case definitions. Each state reported all violent deaths of residents that occurred within the state and those of nonresidents for whom a fatal injury occurred within the state (i.e., occurrent deaths). Once a violent death was identified, NVDRS data abstractors linked source documents, linked deaths within each incident, coded data elements, and wrote short narratives of the incident. State-level data were then consolidated and analyzed for this aggregate report.

Numbers, percentages, and crude rates are presented in aggregate for all deaths by abstractor-assigned manner of death. Rates for cells with frequency <20 are not reported because of the instability of those rates (*8*). Rates could not be calculated for some variables (e.g., marital status and precipitating circumstances) because denominators were unknown. Bridged-race 2014 population estimates were used as denominators in the crude rate calculations (*9*). For compatible numerators for rate calculations to be derived, records listing multiple races were recoded to a single race, when possible, using race-bridging methods described by NCHS (https://www.cdc.gov/nchs/nvss/bridged_race.htm).

## Results

### All Deaths Captured by NVDRS

#### Deaths by Manner, Method, and Location

The 18 NVDRS states included in this report collected data concerning 22,098 incidents and 22,618 deaths that occurred in 2014. The crude death rate was 21.2 deaths per 100,000 population. Suicides (n = 14,834 [65.6%]) accounted for the highest rate of violent deaths (13.9 per 100,000 population), followed by homicides (n = 5,100 [22.5%]) (4.8 per 100,000 population). Deaths of undetermined intent (n = 2,257 [10.0%]), legal intervention deaths (n = 283 [1.3%]), and unintentional firearm deaths (n = 144 [<1.0%]) occurred at lower rates (2.1, 0.3, and 0.1 per 100,000 population, respectively). Firearms were the method used in 51.1% of deaths, hanging/strangulation/suffocation in 18.8%, and poisoning in 17.4% (rates: 10.8, 4.0, and 3.7 per 100,000 population, respectively). Rates for all other methods were lower. For all deaths, a house or apartment was the most common location where injury occurred (69.5%), followed by a street or highway (6.3%), a motor vehicle (4.9%), or a natural area (4.0%) (Table 1).

**TABLE 1:** Number,* percentage,^†^ and rate^§^ of deaths, by incident type, manner of death, method used and location in which injury occurred — National Violent Death Reporting System, 18 states,^¶^ 2014

Characteristic	No. (%)	Rate
**Incident type**		
Suicide, single	14,560 (65.9)	13.7
Homicide, single	4,459 (20.2)	4.2
Undetermined intent, single	2,222 (10.1)	2.1
Unintentional firearm, single	144 (<1.0)	0.1
Suicide, multiple	23 (<1.0)	**
Homicide, multiple	169 (<1.0)	**
Undetermined intent, multiple	12 (<1.0)	**
Legal intervention,^††^ single/multiple	281 (1.3)	**
Homicide followed by suicide	222 (1.0)	**
Other combinations of deaths	6 (<1.0)	**
**Total**	**22,098 (100)**	**20.7**
**Manner of death**		
Suicide or intentional self-harm	14,834 (65.6)	13.9
Homicide	5,100 (22.5)	4.8
Undetermined intent	2,257 (10.0)	2.1
Legal Intervention^††^	283 (1.3)	0.3
Unintentional firearm	144 (<1.0)	0.1
**Total**	**22,618 (100)**	**21.2**
**Method**		
Firearm	11,554 (51.1)	10.8
Hanging/strangulation/suffocation	4,243 (18.8)	4.0
Poisoning	3,946 (17.4)	3.7
Sharp instrument	955 (4.2)	0.9
Blunt instrument	334 (1.5)	0.3
Fall	320 (1.4)	0.3
Motor vehicle (e.g., car, bus, motorcycle, or other transport vehicle)	269 (1.2)	0.3
Personal weapons (e.g., hands, feet, fists)	255 (1.1)	0.2
Drowning	214 (<1.0)	0.2
Fire/burns	134 (<1.0)	0.1
Intentional neglect	22 (<1.0)	0.0
Other (single method)	75 (<1.0)	0.1
Unknown	297 (1.3)	0.3
**Total**	**22,618 (100)**	**21.2**
**Location**		
House, apartment	15,713 (69.5)	14.7
Street/highway	1,420 (6.3)	1.3
Motor vehicle	1,105 (4.9)	1.0
Natural area	916 (4.0)	0.9
Hotel/motel	509 (2.3)	0.5
Parking lot/public garage/public transport	404 (1.8)	0.4
Commercial/retail area	303 (1.3)	0.3
Jail/prison	258 (1.1)	0.2
Park, playground, sports/athletic area	238 (1.1)	0.2
Bar/nightclub	118 (<1.0)	0.1
Supervised residential facility	98 (<1.0)	0.1
Railroad tracks	94 (<1.0)	0.1
Hospital or medical facility	78 (<1.0)	0.1
Farm	69 (<1.0)	0.1
Abandoned house/building/warehouse	64 (<1.0)	0.1
Industrial or construction area	58 (<1.0)	0.1
Preschool/school/college/school bus	56 (<1.0)	0.1
Office building	38 (<1.0)	0.0
Other	370 (1.6)	0.3
Unknown	709 (3.1)	0.7
**Total**	**22,618 (100)**	**21.2**

* No. incidents = 22,098; no. decedents = 22,368 (98.9%); no. suspect/decedents = 250 (1.1%). The Incident type characteristic reports number of incidents; all others report number of deaths.

^†^ Percentages might not total 100% due to rounding.

^§^ Per 100,000 population.

^¶^ Alaska, Colorado, Georgia, Kentucky, Maryland, Massachusetts, Michigan, North Carolina, New Jersey, New Mexico, Ohio, Oklahoma, Oregon, Rhode Island, South Carolina, Utah, Virginia, and Wisconsin.

** Because the number of decedents varies in incidents involving multiple deaths, numerators cannot be determined to compute rates.

^††^ The term “legal intervention” does not denote the lawfulness or legality of the circumstances surrounding the death.

#### Substance Use

Poisoning and substance abuse figure prominently in violent deaths. In 2014, poisoning was the third most common means of suicide captured by NVDRS overall and was the top suicide method for women. In addition, poisoning deaths constitute the overwhelming majority of deaths of undetermined intent captured by NVDRS (73.8%).

Approximately 75% of all decedents who received toxicology testing had one or more substances detected, and 42.8% had two or more (range: two–12). These percentages were even higher for decedents with a history of drug or alcohol abuse (i.e., substance abuse): of those decedents, 87.8% had one or more substances detected in their system at the time of death, and 53.3% had more than one.

The percentage of suicide decedents with prior substance abuse problems (other than alcohol) who died by poisoning was twice as high as the percentage of suicide decedents without prior substance abuse problems who died by poisoning (26% versus 13%, respectively). The most common substance types detected in suicide decedents with a history of substance abuse were alcohol (51.1%), over-the-counter or otherwise unclassified drugs (31.7%), benzodiazepines (23.3%), opioids (23.2%), antidepressants (20%), and marijuana (13.6%).

Toxicology results also reveal the substances that were not only present in the systems of decedents across all manners but also identify those that directly caused the person’s death. The substances that caused the largest percentage of poisoning deaths across all manners of deaths were opioids (38%), over-the-counter or otherwise unclassified drugs (29%), benzodiazepines (19%), antidepressants (19%), alcohol (15%), and carbon monoxide (10%). The most frequently co-occurring substances causing death across all manners of poisoning deaths were benzodiazepines/opioids (28%), antidepressants/ benzodiazepines (15%), alcohol/opioids (14%), antidepressants/opioids (14%), alcohol/benzodiazepines (11%), and alcohol/antidepressants (9%). For suicides overall, the substances that most frequently caused death were over-the-counter or otherwise unclassified drugs (35%), opioids (27%), antidepressants (23%), benzodiazepines (20%), carbon monoxide (15%), and alcohol (13%).

### Suicides

#### Sex, Race/Ethnicity, Age Group, and Marital Status

The 18 NVDRS states included in this report collected data for 2014 concerning 14,805 suicide incidents, which included 14,834 deaths (Table 2). Overall, the crude suicide rate was 13.9 per 100,000 population. The rate for males was more than three times the rate for females (21.9 and 6.3 per 100,000 population, respectively). Non-Hispanic American Indian/Alaska Natives and non-Hispanic whites had the highest rates of suicide (19.1 and 17.0 per 100,000 population, respectively). The highest rates of suicide by age group occurred among persons aged 45–54 years and 55–64 years (20.4 and 18.9 per 100,000 population, respectively). Persons aged 10–14 years had the lowest rate of suicide among all age groups (2.4 per 100,000 population).

**TABLE 2:** Number, percentage,* and rate^†^ of suicides, by decedent's sex, age group, race/ethnicity, and marital status — National Violent Death Reporting System, 18 states,^§^ 2014

	Male		Female		Total	
Characteristic	No. (%)	Rate	No. (%)	Rate	No. (%)	Rate
**Age group (yrs)**						
<10	^¶^	^¶^	^¶^	^¶^	**^¶^**	**^¶^**
10–14	117 (1.0)	3.3	53 (1.6)	1.6	**170 (1.1)**	**2.4**
15–19	518 (4.5)	14.3	172 (5.1)	5.0	**690 (4.7)**	**9.8**
20–24	966 (8.4)	24.7	203 (6.0)	5.5	**1,169 (7.9)**	**15.3**
25–29	892 (7.8)	24.7	218 (6.4)	6.2	**1,110 (7.5)**	**15.5**
30–34	925 (8.1)	26.3	246 (7.2)	7.0	**1,171 (7.9)**	**16.6**
35–44	1,797 (15.7)	26.8	626 (18.4)	9.2	**2,423 (16.3)**	**17.9**
45–54	2,178 (19.0)	30.1	830 (24.4)	11.0	**3,008 (20.3)**	**20.4**
55–64	1,953 (17.1)	29.7	635 (18.7)	9.0	**2,588 (17.4)**	**18.9**
65–74	1,107 (9.7)	26.6	257 (7.6)	5.4	**1,364 (9.2)**	**15.3**
75–84	671 (5.9)	35.1	119 (3.5)	4.6	**790 (5.3)**	**17.7**
≥85	313 (2.7)	47.2	36 (1.1)	2.7	**349 (2.4)**	**17.5**
Unknown	1 (<1.0)	**	0 (0.0)	**	**1 (<1.0)**	**
**Total**	**11,438 (100)**	**21.9**	**3,396 (100)**	**6.3**	**14,834 (100)**	**13.9**
**Race/ethnicity**						
White, non-Hispanic	9,626 (84.2)	26.6	2,880 (84.8)	7.7	**12,506 (84.3)**	**17.0**
Black, non-Hispanic	767 (6.7)	9.8	200 (5.9)	2.3	**967 (6.5)**	**5.9**
American Indian/Alaska Native, non-Hispanic	182 (1.6)	30.7	49 (1.4)	8.0	**231 (1.6)**	**19.1**
Asian/Pacific Islander	199 (1.7)	9.2	86 (2.5)	3.7	**285 (1.9)**	**6.3**
Hispanic^††^	555 (4.9)	10.0	147 (4.3)	2.8	**702 (4.7)**	**6.5**
Other	71 (<1.0)	**	14 (<1.0)	**	**85 (<1.0)**	**
Unknown	38 (<1.0)	**	20 (<1.0)	**	**58 (<1.0)**	**
**Total**	**11,438 (100)**	**21.9**	**3,396 (100)**	**6.3**	**14,834 (100)**	**13.9**
**Marital status^§§^**						
Married	3,819 (34.5)	^¶¶^	1,105 (34.2)	^¶¶^	**4,924 (34.4)**	**^¶¶^**
Never married	3,669 (33.1)	^¶¶^	769 (23.8)	^¶¶^	**4,438 (31.0)**	**^¶¶^**
Widowed	608 (5.5)	^¶¶^	254 (7.9)	^¶¶^	**862 (6.0)**	**^¶¶^**
Divorced	2,317 (20.9)	^¶¶^	953 (29.5)	^¶¶^	**3,270 (22.8)**	**^¶¶^**
Married, but separated	367 (3.3)	^¶¶^	79 (2.4)	^¶¶^	**446 (3.1)**	**^¶¶^**
Single, not otherwise specified	171 (1.5)	^¶¶^	41 (1.3)	^¶¶^	**212 (1.5)**	**^¶¶^**
Unknown	131 (1.2)	^¶¶^	34 (1.1)	^¶¶^	**165 (1.2)**	**^¶¶^**
**Total**	**11,082 (100)**	**^¶¶^**	**3,235 (100)**	**^¶¶^**	**14,317 (100)**	**^¶¶^**

* Percentages might not total 100% due to rounding.

^†^ Per 100,000 population.

^§^ Alaska, Colorado, Georgia, Kentucky, Maryland, Massachusetts, Michigan, North Carolina, New Jersey, New Mexico, Ohio, Oklahoma, Oregon, Rhode Island, South Carolina, Utah, Virginia, and Wisconsin.

^¶^ Suicide is not reported for decedents aged <10 years, as per standard in the suicide prevention literature.

** Rates not reported when number of decedents is <20 or when race/ethnicity or age categories are “Other” or “Unknown.”

^††^ Includes persons of any race.

^§§^ Includes decedents aged ≥18 years only.

^¶¶^ Rates cannot be computed for marital status because denominators are unknown.

Decedents aged 35–64 years accounted for more than half (51.8%) of suicides among males. Rates among males were highest for men aged ≥85 years, followed by men aged 75–84 and 45–54 years (47.2, 35.1, and 30.1 per 100,000 population, respectively). Non-Hispanic American Indian/Alaska Natives (30.7 per 100,000 population) and non-Hispanic whites (26.6 per 100,000 population) had the highest rates of any male subgroups; these rates were approximately three times the rate for males with the lowest rate, Asian/Pacific Islanders (9.2 per 100,000 population). Decedents aged 35–64 years also accounted for the majority (61.5%) of suicides among females. The rate among females was highest for women aged 45–54 years (11.0 per 100,000). Non-Hispanic American Indian/Alaska Natives (8.0 per 100,000 population) and non-Hispanic whites (7.7 per 100,000 population) had the highest suicide rates among females; rates were lowest among non-Hispanic black (2.3 per 100,000 population) and Hispanic (2.8 per 100,000 population) females. Of suicide decedents aged ≥18 years, 34.4% were married, 31.0% had never been married, and 22.8% were divorced at the time of death (Table 2).

#### Method and Location of Injury

Firearms were used in more than half of suicides (51.1%), followed by hanging/strangulation/suffocation (27.0%) and poisoning (15.2%) (rates: 7.1, 3.8, and 2.1 per 100,000 population, respectively) (Table 3). Among males, the most common method used was a firearm (56.6%), followed by hanging/strangulation/suffocation (27.0%) (Table 3). Among females, poisoning was the most common method used (33.4%), followed by a firearm (32.6%). The most common place of suicide was a house or apartment (75.3%), followed by a natural area (4.6%), a motor vehicle (4.4%), a hotel/motel (2.4%), and a street/highway (2.3%).

**TABLE 3:** Number, percentage, * and rate^†^ of suicides, by decedent's sex, method used, and location in which injury occurred — National Violent Death Reporting System, 18 states,^§^ 2014

	Male	Female	Total	
Characteristic	No. (%)	No. (%)	No. (%)	Rate
**Method**				
Firearm	6,469 (56.6)	1,106 (32.6)	**7,575 (51.1)**	**7.1**
Hanging/strangulation/suffocation	3,091 (27.0)	907 (26.7)	**3,998 (27.0)**	**3.8**
Poisoning	1,125 (9.8)	1,134 (33.4)	**2,259 (15.2)**	**2.1**
Fall	206 (1.8)	59 (1.7)	**265 (1.8)**	**0.2**
Sharp instrument	210 (1.8)	54 (1.6)	**264 (1.8)**	**0.2**
Motor vehicles (e.g., car, bus, motorcycle, or other transport vehicle)	146 (1.3)	48 (1.4)	**194 (1.3)**	**0.2**
Drowning	83 (<1.0)	49 (1.4)	**132 (<1.0)**	**0.1**
Fire/burns	39 (<1.0)	17 (<1.0)	**56 (<1.0)**	**0.1**
Other (single method)	23 (<1.0)	5 (<1.0)	**28 (<1.0)**	**0.0**
Unknown	32 (<1.0)	12 (<1.0)	**44 (<1.0)**	**0.0**
**Total**	**11,438 (100)**	**3,396 (100)**	**14,834 (100)**	**13.9**
**Location**				
House, apartment	8,453 (73.9)	2,716 (80.0)	**11,169 (75.3)**	**10.5**
Natural area	573 (5.0)	113 (3.3)	**686 (4.6)**	**0.6**
Motor vehicle	528 (4.6)	129 (3.8)	**657 (4.4)**	**0.6**
Hotel/motel	248 (2.2)	108 (3.2)	**356 (2.4)**	**0.3**
Street/highway	282 (2.5)	52 (1.5)	**334 (2.3)**	**0.3**
Jail/prison	195 (1.7)	22 (<1.0)	**217 (1.5)**	**0.2**
Park, playground, sports/athletic area	158 (1.4)	19 (<1.0)	**177 (1.2)**	**0.2**
Parking lot/public garage/public transport	140 (1.2)	33 (<1.0)	**173 (1.2)**	**0.2**
Commercial/retail area	96 (<1.0)	13 (<1.0)	**109 (<1.0)**	**0.1**
Railroad tracks	59 (<1.0)	18 (<1.0)	**77 (<1.0)**	**0.1**
Farm	59 (<1.0)	2 (<1.0)	**61 (<1.0)**	**0.1**
Hospital or medical facility	39 (<1.0)	17 (<1.0)	**56 (<1.0)**	**0.1**
Supervised residential facility	40 (<1.0)	16 (<1.0)	**56 (<1.0)**	**0.1**
Preschool/school/college/school bus	40 (<1.0)	6 (<1.0)	**46 (<1.0)**	**0.0**
Industrial or construction area	38 (<1.0)	0 (0.0)	**38 (<1.0)**	**0.0**
Office building	26 (<1.0)	4 (<1.0)	**30 (<1.0)**	**0.0**
Other	223 (1.9)	56 (1.6)	**279 (1.9)**	**0.3**
Unknown	222 (1.9)	70 (2.1)	**292 (2.0)**	**0.3**
**Total**	**11,438 (100)**	**3,396 (100)**	**14,834 (100)**	**13.9**

* Percentages might not total 100% due to rounding.

^†^ Per 100,000 population.

^§^ Alaska, Colorado, Georgia, Kentucky, Maryland, Massachusetts, Michigan, North Carolina, New Jersey, New Mexico, Ohio, Oklahoma, Oregon, Rhode Island, South Carolina, Utah, Virginia, and Wisconsin.

#### Toxicology Results of Decedent

Tests for alcohol were conducted for 53.1% of suicide decedents. Tests for amphetamines, antidepressants, benzodiazepines, cocaine, marijuana, and opiates were conducted for 31.1%, 26.0%, 33.7%, 32.6%, 29.0%, and 36.2%, respectively (Table 4). Among those with positive results for alcohol (40.2%), 69.4% had a blood alcohol concentration (BAC) ≥0.08 g/dL. Opiates (including heroin and prescription pain medications) were identified in 30.0% of decedents tested for these substances; cocaine and marijuana were identified in 5.7% and 21.0% of decedents tested, respectively. Of those tested for antidepressants, 40.8% had positive results at the time of their death, and 32.6% of those tested for benzodiazepines had positive results (Table 4).

**TABLE 4:** Number* and percentage of suicide decedents who were tested for alcohol and drugs whose results were positive,^†^ by toxicology variable — National Violent Death Reporting System, 18 states,^§^ 2014

	Tested	Positive
Toxicology variable	No. (%)	No. (%)
BAC^¶^	7,883 (53.1)	3,168 (40.2)
Alcohol <0.08 g/dL		874 (27.6)
Alcohol ≥0.08 g/dL		2,200 (69.4)
Alcohol positive, level unknown		94 (3.0)
Amphetamines	4,619 (31.1)	398 (8.6)
Anticonvulsants	3,126 (21.1)	457 (14.6)
Antidepressants	3,862 (26.0)	1,577 (40.8)
Antipsychotics	3,072 (20.7)	308 (10.0)
Barbiturates	3,748 (25.3)	119 (3.2)
Benzodiazepines	4,996 (33.7)	1,631 (32.6)
Carbon monoxide	1,805 (12.2)	354 (19.6)
Cocaine	4,837 (32.6)	274 (5.7)
Marijuana	4,309 (29.0)	903 (21.0)
Muscle relaxants	3,111 (21.0)	245 (7.9)
Opiates	5,368 (36.2)	1,611 (30.0)
Other drugs/substances**	2,654 (17.9)	2,589 (97.6)

**Abbreviation:** BAC = blood alcohol concentration.

* No. of suicide decedents = 14,834.

^†^ Percentage is of decedents tested for toxicology variable.

^§^ Alaska, Colorado, Georgia, Kentucky, Maryland, Massachusetts, Michigan, North Carolina, New Jersey, New Mexico, Ohio, Oklahoma, Oregon, Rhode Island, South Carolina, Utah, Virginia, and Wisconsin.

^¶^ BAC of ≥0.08 g/dL is over the legal limit in all states and is used as the standard for intoxication.

** Other drugs/substances indicated if any results were positive; levels for these drugs/substances are not measured.

#### Precipitating Circumstances

Precipitating circumstances were known for 90.1% of suicides. Overall, mental health problems were the most common circumstance; 36.6% of decedents were described as experiencing a depressed mood at the time of their death, 47.7% as having a current diagnosed mental health problem, and 28.3% as receiving mental health treatment (Table 5). Among the 6,375 decedents with a current diagnosed mental health problem, depression/dysthymia (73.3%), anxiety disorder (15.9%), and bipolar disorder (15.0%) were the most common diagnoses for both males and females (Table 6).

**TABLE 5:** Number* and percentage^†^ of suicides, by precipitating circumstances and decedent's sex — National Violent Death Reporting System, 18 states,^§^ 2014

	Male	Female	Total
Precipitating circumstances	No. (%)	No. (%)	No. (%)
**Mental health/substance abuse**			
Current diagnosed mental health problem	4,427 (43.2)	1,948 (62.3)	**6,375 (47.7)**
Current depressed mood	3,747 (36.6)	1,142 (36.5)	**4,889 (36.6)**
History of ever being treated for a mental health problem	3,291 (32.1)	1,529 (48.9)	**4,820 (36.1)**
Current mental health treatment	2,509 (24.5)	1,275 (40.8)	**3,784 (28.3)**
Alcohol problem	1,903 (18.6)	488 (15.6)	**2,391 (17.9)**
Other substance abuse problem (excludes alcohol)	1,521 (14.9)	603 (19.3)	**2,124 (15.9)**
Other addiction (e.g., gambling or sexual)	57 (<1.0)	24 (<1.0)	**81 (<1.0)**
**Interpersonal**			
Intimate partner problem	2,957 (28.9)	823 (26.3)	**3,780 (28.3)**
Family relationship problem	945 (9.2)	390 (12.5)	**1,335 (10.0)**
Other death of family member or friend within past 5 years	575 (5.6)	227 (7.3)	**802 (6.0)**
Perpetrator of interpersonal violence within past month	322 (3.1)	28 (<1.0)	**350 (2.6)**
Other relationship problem (nonintimate)	224 (2.2)	60 (1.9)	**284 (2.1)**
Suicide of family member or friend within past 5 years	197 (1.9)	83 (2.7)	**280 (2.1)**
Victim of interpersonal violence within past month	23 (<1.0)	28 (<1.0)	**51 (<1.0)**
**Life stressor**			
Crisis within previous or upcoming 2 weeks	3,687 (36.0)	902 (28.8)	**4,589 (34.3)**
Physical health problem	2,294 (22.4)	642 (20.5)	**2,936 (22.0)**
Argument or conflict	1,542 (15.1)	500 (16.0)	**2,042 (15.3)**
Job problem	1,259 (12.3)	227 (7.3)	**1,486 (11.1)**
Financial problem	1,074 (10.5)	240 (7.7)	**1,314 (9.8)**
Recent criminal legal problem	1,091 (10.7)	128 (4.1)	**1,219 (9.1)**
Eviction or loss of home	369 (3.6)	96 (3.1)	**465 (3.5)**
Noncriminal legal problem	329 (3.2)	74 (2.4)	**403 (3.0)**
School problem	146 (1.4)	53 (1.7)	**199 (1.5)**
History of child abuse/neglect	81 (<1.0)	78 (2.5)	**159 (1.2)**
Traumatic anniversary	57 (<1.0)	26 (<1.0)	**83 (<1.0)**
Physical fight (two persons, not a brawl)	63 (<1.0)	11 (<1.0)	**74 (<1.0)**
Caretaker abuse/neglect led to suicide	4 (<1.0)	8 (<1.0)	**12 (<1.0)**
Exposure to disaster	12 (<1.0)	0 (0.0)	**12 (<1.0)**
**Crime and criminal activity**			
Precipitated by another crime	414 (4.0)	34 (1.1)	**448 (3.4)**
Crime in progress^¶^	99 (23.9)	9 (26.5)	**108 (24.1)**
Terrorist attack	0 (0.0)	0 (0.0)	**0 (0.0)**
**Suicide event**			
Left a suicide note	3,240 (31.6)	1,269 (40.6)	**4,509 (33.7)**
History of suicidal thoughts or plans	3,121 (30.5)	1,097 (35.1)	**4,218 (31.6)**
History of suicide attempt(s)	1,645 (16.1)	999 (31.9)	**2,644 (19.8)**
**Suicide disclosure**			
Disclosed suicide intent	2,573 (25.1)	717 (22.9)	**3,290 (24.6)**
Disclosed intent to whom**			
Previous or current intimate partner	1,028 (40.0)	239 (33.3)	**1,267 (38.5)**
Other family member	754 (29.3)	224 (31.2)	**978 (29.7)**
Friend/colleague	290 (11.3)	105 (14.6)	**395 (12.0)**
Health care worker	90 (3.5)	44 (6.1)	**134 (4.1)**
Neighbor	42 (1.6)	14 (2.0)	**56 (1.7)**
Other person	222 (8.6)	38 (5.3)	**260 (7.9)**
Unknown	147 (5.7)	53 (7.4)	**200 (6.1)**
**Total suicides with precipitating circumstances**	**10,241 (100)**	**3,127 (100)**	**13,368 (100)**

* Includes suicides with one or more precipitating circumstances. Circumstances were unknown for 1,466 decedents (1,197 males and 269 females). Numbers do not equal the sums of the columns because more than one circumstance could have been present per decedent.

^†^ Denominator includes only those suicides with one or more precipitating circumstances. The sum of percentages in columns exceeds 100% because more than one circumstance could have been present per decedent.

^§^ Alaska, Colorado, Georgia, Kentucky, Maryland, Massachusetts, Michigan, North Carolina, New Jersey, New Mexico, Ohio, Oklahoma, Oregon, Rhode Island, South Carolina, Utah, Virginia, and Wisconsin.

^¶^ Denominator includes only those decedents involved in an incident that was precipitated by another crime. Suicide deaths precipitated by this circumstance include situations such as when the suicide victim also committed a homicide or attempted homicide.

** Denominator is decedents who disclosed intent.

**TABLE 6:** Number* and percentage^†^ of suicide decedents with a current mental health problem, by diagnosis — National Violent Death Reporting System, 18 states,^§^ 2014

	Male	Female	Total
Mental health problem	No. (%)	No. (%)	No. (%)
Depression/dysthymia	3,207 (72.4)	1,469 (75.4)	**4,676 (73.3)**
Anxiety disorder	652 (14.7)	361 (18.5)	**1,013 (15.9)**
Bipolar disorder	574 (13.0)	383 (19.7)	**957 (15.0)**
PTSD	251 (5.7)	55 (2.8)	**306 (4.8)**
Schizophrenia	231 (5.2)	71 (3.6)	**302 (4.7)**
ADD/ADHD	128 (2.9)	31 (1.6)	**159 (2.5)**
OCD	29 (<1.0)	10 (<1.0)	**39 (<1.0)**
Eating disorder	4 (<1.0)	21 (1.1)	**25 (<1.0)**
Other	227 (5.1)	106 (5.4)	**333 (5.2)**
Unknown	431 (9.7)	161 (8.3)	**592 (9.3)**
**Total decedents with a current diagnosed mental health problem**	**4,427 (100)**	**1,948 (100)**	**6,375 (100)**
			

**Abbreviation:** ADD/ADHD = attention deficit disorder/attention deficit hyperactivity disorder; OCD = obsessive-compulsive disorder; PTSD = posttraumatic stress disorder.

* Includes decedents with one or more diagnosed mental health problem. Numbers do not equal the sum of the column because more than one diagnosis could have been present per decedent.

^†^ Denominator includes only those decedents with one or more diagnosed mental health problem. Sums of percentages in columns exceed 100% because decedents could have had more than one diagnosis.

^§^ Alaska, Colorado, Georgia, Kentucky, Maryland, Massachusetts, Michigan, North Carolina, New Jersey, New Mexico, Ohio, Oklahoma, Oregon, Rhode Island, South Carolina, Utah, Virginia, and Wisconsin.

Equivalent percentages of both male (36.6%) and female (36.5%) decedents were reported to have a depressed mood at the time of their suicide. Greater percentages of females were reported to have a current diagnosed mental health problem (62.3% of females and 43.2% of males) and/or to be receiving current mental health treatment (40.8% of females and 24.5% of males) (Table 5).

Among 13,368 suicides with known circumstances, 33.7% of decedents left a suicide note, 31.6% had a history of suicidal thoughts or plans, 19.8% had a history of previous suicide attempts, and 24.6% had disclosed suicidal intent to another person (Table 5). Of those who disclosed intent, the majority of disclosures were to a previous or current intimate partner (38.5%) or to some other family member (29.7%). Alcohol or other substance abuse problems were indicated for 17.9% and 15.9% of suicide decedents, respectively. A higher percentage of males (18.6% of males and 15.6% of females) reportedly had alcohol problems, and a higher percentage of females had another substance problem indicated (19.3% of females and 14.9% of males). Other common circumstances were a crisis in the preceding or upcoming 2 weeks (34.3%) and intimate partner problems (28.3%). Physical health problems (22.0%), an argument or conflict (15.3%), job or financial problems (11.1% and 9.8%, respectively), family relationship problems (10.0%), and recent criminal legal problems (9.1%) were also reported to have preceded suicides.

Job problems were noted as a precipitating circumstance in a higher percentage of males than females (12.3% and 7.3%, respectively), as were financial problems (10.5% and 7.7%) and recent criminal legal problems (10.7% and 4.1%). In contrast, family (nonintimate partner) relationship problems were a precipitating circumstance in a higher percentage of suicides of females than males (12.5% and 9.2%, respectively). Although indicated in a small percentage of suicides, males were more often a perpetrator of interpersonal violence in the month before death (3.1%) than were females (<1.0%) (Table 5).

### Homicides

#### Sex, Race/Ethnicity, Age Group, and Marital Status

The 18 NVDRS states included in this report collected data concerning 4,850 homicide incidents, which included 5,100 deaths in 2014 (Table 7). Overall, the crude homicide rate was 4.8 deaths per 100,000 population. The majority of homicide decedents aged ≥18 years (61.2%) had never been married, 19.1% were married, and 12.6% were divorced at the time of their death (Table 8). In more than half (51.3%) of homicides, the relationship of the victim to the suspect was not known; when the relationship was known, the suspect most often was an acquaintance or friend (28.1%), a spouse or intimate partner (22.6%), or a stranger (13.6%).

**TABLE 7:** Number, percentage,* and rate^†^ of homicides, by decedent's sex, age group, and race/ethnicity — National Violent Death Reporting System, 18 states,^§^ 2014

	Male		Female		Total	
Characteristic	No. (%)	Rate	No. (%)	Rate	No. (%)	Rate
**Age group (yrs)**						
<1	44 (1.1)	6.6	35 (3.0)	5.5	**79 (1.5)**	**6.1**
1–4	67 (1.7)	2.5	50 (4.3)	1.9	**118 (2.3)**	**2.2**
5–9	21 (<1.0)	0.6	19 (1.6)	^¶^	**40 (<1.0)**	**0.6**
10–14	37 (<1.0)	1.0	20 (1.7)	0.6	**57 (1.1)**	**0.8**
15–19	311 (7.9)	8.6	58 (5.0)	1.7	**369 (7.2)**	**5.2**
20–24	805 (20.4)	20.6	126 (10.9)	3.4	**931 (18.3)**	**12.2**
25–29	660 (16.7)	18.2	117 (10.1)	3.3	**777 (15.2)**	**10.9**
30–34	476 (12.1)	13.5	127 (11.0)	3.6	**603 (11.8)**	**8.6**
35–44	598 (15.2)	8.9	199 (17.2)	2.9	**797 (15.6)**	**5.9**
45–54	471 (11.9)	6.5	168 (14.6)	2.2	**639 (12.5)**	**4.3**
55–64	261 (6.6)	4.0	104 (9.0)	1.5	**365 (7.2)**	**2.7**
65–74	129 (3.3)	3.1	62 (5.4)	1.3	**191 (3.7)**	**2.1**
75–84	51 (1.3)	2.7	44 (3.8)	1.7	**95 (1.9)**	**2.1**
≥85	10 (<1.0)	^¶^	24 (2.1)	1.8	**34 (<1.0)**	**1.7**
Unknown	4 (<1.0)	^¶^	1 (<1.0)	^¶^	**5 (<1.0)**	**^¶^**
**Total**	**3,945 (100)**	**7.5**	**1,154 (100)**	**2.1**	**5,100 (100)**	**4.8**
**Race/ethnicity**						
White, non-Hispanic	1,006 (25.5)	2.8	573 (49.7)	1.5	**1,580 (31.0)**	**2.1**
Black, non-Hispanic	2,420 (61.3)	31.0	412 (35.7)	4.8	**2,832 (55.5)**	**17.3**
American Indian/Alaska Native, non-Hispanic	85 (2.2)	14.4	28 (2.4)	4.6	**113 (2.2)**	**9.4**
Asian/Pacific Islander	38 (<1.0)	1.8	21 (1.8)	0.9	**59 (1.2)**	**1.3**
Hispanic**	358 (9.1)	6.4	107 (9.3)	2.0	**465 (9.1)**	**4.3**
Other	23 (<1.0)	^¶^	5 (<1.0)	^¶^	**28 (<1.0)**	**^¶^**
Unknown	15 (<1.0)	^¶^	8 (<1.0)	^¶^	**23 (<1.0)**	**^¶^**
**Total**	**3,945 (100)**	**7.5**	**1,154 (100)**	**2.1**	**5,100 (100)**	**4.8**

* Percentages might not total 100% due to rounding. Sex was unknown for one decedent.

^†^ Per 100,000 population.

^§^ Alaska, Colorado, Georgia, Kentucky, Maryland, Massachusetts, Michigan, North Carolina, New Jersey, New Mexico, Ohio, Oklahoma, Oregon, Rhode Island, South Carolina, Utah, Virginia, and Wisconsin.

^¶^ Rate is not reported when number of decedents is <20 or when age or race/ethnicity is other or unknown.

** Includes persons of any race.

**TABLE 8:** Number* and percentage^†^ of homicides, by decedent's marital status and victim-suspect relationship^§^ — National Violent Death Reporting System, 18 states, ^¶^ 2014

Characteristic	No. (%)
Marital status**	
Never married	2,816 (61.2)
Married	877 (19.1)
Divorced	578 (12.6)
Widowed	148 (3.2)
Single, not otherwise specified	111 (2.4)
Married, but separated	68 (1.5)
**Total**	**4,598 (100)**
**Relationship**	
Acquaintance/friend	698 (28.1)
Spouse/intimate partner (current or former)	561 (22.6)
Stranger	337 (13.6)
Other relative	189 (7.6)
Child	154 (6.2)
Parent	139 (5.6)
Other intimate partner involvement**^††^**	58 (2.3)
Rival gang member	15 (<1.0)
Victim was law enforcement officer injured in the line of duty	9 (<1.0)
Other person known to victim	326 (13.1)
Victim injured by a law enforcement officer	0 (0.0)
**Total**	**2,486 (100)**

* No. deaths = 5,100. Marital status was unknown for 76 decedents. Victim-suspect relationship unknown for 2,614 decedents.

^†^ Percentages might not total 100% due to rounding.

^§^ A victim is a person whose death resulted from a violence-related injury; a suspect is a person believed to have inflicted a fatal injury.

^¶^ Alaska, Colorado, Georgia, Kentucky, Maryland, Massachusetts, Michigan, North Carolina, New Jersey, New Mexico, Ohio, Oklahoma, Oregon, Rhode Island, South Carolina, Utah, Virginia, and Wisconsin.

** Includes decedents aged ≥18 years only.

^††^ Death was due to intimate partner-related violence but not between the intimate partners (e.g., child killed by mother’s boyfriend).

The homicide rate for males was approximately three times the rate for females (7.5 and 2.1 per 100,000 population, respectively) (Table 7). Non-Hispanic blacks accounted for more than half (55.5%) of homicides and had the highest rate (17.3 per 100,000 population), followed by non-Hispanic American Indian/Alaska Natives (9.4 per 100,000 population) and Hispanics (4.3 per 100,000 population). Non-Hispanic black males had the highest rate of homicide deaths among males of any race/ethnicity (31.0 per 100,000 population), over 11 times the homicide rate of non-Hispanic white males (2.8 per 100,000). Age-specific homicide rates were highest among persons aged 20–24 years (12.2 per 100,000 population), followed by persons aged 25–29 years (10.9 per 100,000 population). The rate for infants aged <1 year was over two and a half times the rate for children aged 1–4 years (6.1 and 2.2 per 100,000 population, respectively). Rates were lowest among persons aged 5–14 years and >55 years. Among males, the majority of homicide decedents (64.4%) were aged 20–44 years; the rate was highest among men aged 20–24 years (20.6 per 100,000 population). Among females, the homicide rate was highest among infants aged <1 year (5.5 per 100,000 population). The rate among male infants aged <1 year was 6.6 per 100,000 population.

#### Method and Location of Injury

Firearms were used in 68.6% of homicides, followed by sharp instruments (13.3%), blunt instruments (5.4%), personal weapons (e.g., hands, feet, or fists) (4.7%), and hanging/strangulation/suffocation (3.6%) (Table 9). No other method was used in more than 1% of homicides. Firearms were the most common method used in homicides of males (73.5%) and females (52.2%) (Table 9). Hanging/strangulation/suffocation was more common among females (8.7%) than males (2.1%), as was use of blunt instruments (8.1% and 4.6%, respectively). A house or apartment was the most common location of homicide (51.8%: males and females 45.9% and 72.0%, respectively), followed by a street or highway (18.6%: males and females 21.9% and 7.5%, respectively), a motor vehicle (7.2%), and a parking lot, a public garage, or public transport (3.7%) (Table 9).

**TABLE 9:** Number, percentage,* and rate^†^ of homicides, by decedent's sex, method used, and location in which injury occurred — National Violent Death Reporting System, 18 states,^§^ 2014

	Male	Female	Total	
Characteristic	No. (%)	No. (%)	No. (%)	Rate
**Method**				
Firearm	2,899 (73.5)	602 (52.2)	**3,501 (68.6)**	**3.3**
Sharp instrument	482 (12.2)	197 (17.1)	**679 (13.3)**	**0.6**
Blunt instrument	180 (4.6)	93 (8.1)	**273 (5.4)**	**0.3**
Personal weapons (e.g., hands, feet, fists)	165 (4.2)	75 (6.5)	**240 (4.7)**	**0.2**
Hanging/strangulation/suffocation	83 (2.1)	100 (8.7)	**184 (3.6)**	**0.2**
Motor vehicle (e.g., car, bus, motorcycle, or other transport vehicle)	21 (<1.0)	14 (1.2)	**35 (<1.0)**	**0.0**
Fire/burns	15 (<1.0)	11 (<1.0)	**26 (<1.0)**	**0.0**
Poisoning	9 (<1.0)	12 (1.0)	**21 (<1.0)**	**0.0**
Intentional neglect	4 (<1.0)	11 (<1.0)	**15 (<1.0)**	**^¶^**
Fall	6 (<1.0)	5 (<1.0)	**11 (<1.0)**	**^¶^**
Drowning	3 (<1.0)	3 (<1.0)	**6 (<1.0)**	**^¶^**
Other (single method)	19 (<1.0)	6 (<1.0)	**25 (<1.0)**	**0.0**
Unknown	59 (1.5)	25 (2.2)	**84 (1.6)**	**0.1**
**Total**	**3,945 (100)**	**1,154 (100)**	**5,100 (100)**	**4.8**
**Location**				
House, apartment	1,812 (45.9)	831 (72.0)	**2,644 (51.8)**	**2.5**
Street/highway	864 (21.9)	86 (7.5)	**950 (18.6)**	**0.9**
Motor vehicle	309 (7.8)	58 (5.0)	**367 (7.2)**	**0.3**
Parking lot/public garage/public transport	169 (4.3)	20 (1.7)	**189 (3.7)**	**0.2**
Commercial/retail area	143 (3.6)	18 (1.6)	**161 (3.2)**	**0.2**
Bar/nightclub	105 (2.7)	7 (<1.0)	**112 (2.2)**	**0.1**
Natural area	83 (2.1)	26 (2.3)	**109 (2.1)**	**0.1**
Hotel/motel	48 (1.2)	26 (2.3)	**74 (1.5)**	**0.1**
Park, playground, sports/athletic area	37 (<1.0)	7 (<1.0)	**44 (<1.0)**	**0.0**
Jail/prison	32 (<1.0)	0 (0.0)	**32 (<1.0)**	**0.0**
Abandoned house/building/warehouse	25 (<1.0)	6 (<1.0)	**31 (<1.0)**	**0.0**
Industrial or construction area	15 (<1.0)	3 (<1.0)	**18 (<1.0)**	**^¶^**
Supervised residential facility	9 (<1.0)	6 (<1.0)	**15 (<1.0)**	**^¶^**
Hospital or medical facility	7 (<1.0)	2 (<1.0)	**9 (<1.0)**	**^¶^**
Preschool/school/college/school bus	6 (<1.0)	1 (<1.0)	**7 (<1.0)**	**^¶^**
Farm	4 (<1.0)	0 (0.0)	**4 (<1.0)**	**^¶^**
Railroad tracks	3 (<1.0)	0 (0.0)	**3 (<1.0)**	**^¶^**
Office building	2 (<1.0)	0 (0.0)	**2 (<1.0)**	**^¶^**
Other	55 (1.4)	8 (<1.0)	**63 (1.2)**	**0.1**
Unknown	217 (5.5)	49 (4.2)	**266 (5.2)**	**0.2**
**Total**	**3,945 (100)**	**1,154 (100)**	**5,100 (100)**	**4.8**

* Percentages might not total 100% due to rounding. Sex was unknown for one decedent.

^†^ Per 100,000 population.

^§^ Alaska, Colorado, Georgia, Kentucky, Maryland, Massachusetts, Michigan, North Carolina, New Jersey, New Mexico, Ohio, Oklahoma, Oregon, Rhode Island, South Carolina, Utah, Virginia, and Wisconsin.

^¶^ Rate is not reported when number of decedents is <20.

#### Precipitating Circumstances

Precipitating circumstances were identified for 79.4% of homicides. More than one in three of those homicides was precipitated by another crime (37.4%) (Table 10); in 56.4% of those cases the crime was in progress at the time of the incident. The type of crime most frequently precipitating the homicide was assault/homicide (51.9%), followed by robbery (32.5%), burglary (11.6%), drug trade** (9.3%), rape/sexual assault (2.7%), arson (2.1%), and motor vehicle theft (1.7%). Other common precipitating circumstances were an argument or conflict (36.2%), a physical fight between two people (12.0%), drug involvement (11.6%), and a substance abuse problem other than alcohol abuse (10.3%). In 18.5% of homicides with known circumstances, intimate partner violence (IPV) was identified as a contributing factor (Table 10).

**TABLE 10:** Number* and percentage^†^ of homicides, by precipitating circumstances and decedent's sex — National Violent Death Reporting System, 18 states,^§^ 2014

	Male	Female	Total
Precipitating circumstances	No. (%)	No. (%)	No. (%)
**Mental health/substance abuse**			
Other substance abuse problem (excludes alcohol)	306 (10.1)	111 (11.0)	**417 (10.3)**
Alcohol problem	134 (4.4)	41 (4.1)	**175 (4.3)**
Current diagnosed mental health problem	106 (3.5)	50 (5.0)	**156 (3.9)**
History of ever being treated for a mental health problem	62 (2.0)	33 (3.3)	**95 (2.3)**
Current mental health treatment	47 (1.5)	25 (2.5)	**72 (1.8)**
Current depressed mood	9 (<1.0)	9 (<1.0)	**18 (<1.0)**
Other addiction (e.g., gambling or sexual)	3 (<1.0)	2 (<1.0)	**5 (<1.0)**
**Interpersonal**			
Intimate partner violence-related	269 (8.8)	480 (47.6)	**749 (18.5)**
Family relationship problem	183 (6.0)	83 (8.2)	**266 (6.6)**
Other relationship problem (nonintimate)	178 (5.9)	35 (3.5)	**213 (5.3)**
Jealousy (lovers’ triangle)	58 (1.9)	48 (4.8)	**106 (2.6)**
Victim of interpersonal violence within past month	27 (<1.0)	44 (4.4)	**71 (1.8)**
Perpetrator of interpersonal violence within past month	37 (1.2)	6 (<1.0)	**43 (1.1)**
**Life stressor**			
Argument or conflict	1,152 (37.9)	312 (30.9)	**1,464 (36.2)**
Physical fight (two persons, not a brawl)	430 (14.1)	54 (5.4)	**484 (12.0)**
Crisis within previous or upcoming 2 weeks	220 (7.2)	98 (9.7)	**318 (7.9)**
History of child abuse/neglect	25 (<1.0)	21 (2.1)	**46 (1.1)**
**Crime and criminal activity**			
Precipitated by another crime	1,228 (40.4)	285 (28.2)	**1,513 (37.4)**
Crime in progress^¶^	711 (57.9)	142 (49.8)	**853 (56.4)**
Drug involvement	418 (13.8)	50 (5.0)	**468 (11.6)**
Gang-related	207 (6.8)	26 (2.6)	**233 (5.8)**
Terrorist attack	0 (0.0)	0 (0.0)	**0 (0.0)**
**Homicide event**			
Caretaker abuse/neglect led to death	104 (3.4)	101 (10.0)	**205 (5.1)**
Victim used a weapon	184 (6.1)	7 (<1.0)	**191 (4.7)**
Drive-by shooting	115 (3.8)	12 (1.2)	**127 (3.1)**
Walk by assault	105 (3.5)	11 (1.1)	**116 (2.9)**
Justifiable self defense	105 (3.5)	3 (<1.0)	**108 (2.7)**
Brawl	94 (3.1)	7 (<1.0)	**101 (2.5)**
Mentally ill suspect	60 (2.0)	41 (4.1)	**101 (2.5)**
Random violence	59 (1.9)	20 (2.0)	**79 (2.0)**
Victim was a bystander	39 (1.3)	28 (2.8)	**67 (1.7)**
Victim was an intervener assisting a crime victim	19 (<1.0)	3 (<1.0)	**22 (<1.0)**
Prostitution	12 (<1.0)	7 (<1.0)	**19 (<1.0)**
Victim was a police officer on duty	11 (<1.0)	0 (0.0)	**11 (<1.0)**
Stalking	3 (<1.0)	6 (<1.0)	**9 (<1.0)**
Mercy killing	0 (0.0)	6 (<1.0)	**6 (<1.0)**
Hate crime	2 (<1.0)	1 (<1.0)	**3 (<1.0)**
**Total homicides with precipitating circumstances**	**3,040 (100)**	**1,009 (100)**	**4,049 (100)**

* Includes homicides with one or more precipitating circumstances. Total numbers do not equal the sums of the columns because more than one circumstance could have been present per decedent. Circumstances were unknown for 1,051 decedents (905 males and 145 females; sex was unknown for one decedent).

^†^ Denominator includes only those homicides with one or more precipitating circumstances. The sum of percentages in columns exceeds 100% because more than one circumstance could have been present per decedent.

^§^ Alaska, Colorado, Georgia, Kentucky, Maryland, Massachusetts, Michigan, North Carolina, New Jersey, New Mexico, Ohio, Oklahoma, Oregon, Rhode Island, South Carolina, Utah, Virginia, and Wisconsin.

^¶^ Denominator includes only those decedents involved in an incident that was precipitated by another crime.

IPV was a precipitating factor in 47.6% of homicides among females but only 8.8% among males. An argument or a conflict was a factor in more homicides among males (37.9%) than among females (30.9%). Physical fights precipitated 14.1% of homicides of males, but only 5.4% of homicides of females. Likewise, drug involvement more commonly precipitated homicides of males, accounting for 13.8% of homicides among males and 5.0% among females. However, a substance abuse problem other than alcohol abuse was implicated as a circumstance present in roughly equal percentages of males and females (10.1% and 11.0%, respectively). Gang-related homicides were also more common among males (6.8%) than females (2.6%). Male decedents also used a weapon during the incident in 6.1% and female decedents in <1% of homicides with known circumstances (Table 10).

### Legal Intervention Deaths

#### Sex, Race/Ethnicity and Age Group

The 18 NVDRS states included in this report collected data concerning 281 legal intervention death incidents, which included 283 deaths in 2014 (Table 11). The vast majority of legal intervention deaths occurred among males (93.2%), with the highest rate among men aged 25–29 years (1.2 per 100,000 population), followed by men aged 30–34 years (1.1 per 100,000), 20–24 years (1.0 per 100,000), and 35–44 years (0.9 per 100,000). Non-Hispanic white males accounted for the highest percentage of legal intervention deaths (48.5%), but non-Hispanic black males had the highest rate (1.0 per 100,000 population), followed by Hispanic males (0.8 per 100,000), two to almost three times the rate for non-Hispanic white males (0.4 per 100,000).

**TABLE 11:** Number, percentage,* and rate^†^ of legal intervention^§^ deaths, by decedent's sex, age group, and race/ethnicity — National Violent Death Reporting System, 18 states,^¶^ 2014

	Male		Female		Total	
Characteristic	No. (%)	Rate	No. (%)	Rate	No. (%)	Rate
**Age group (yrs)**						
<1	0 (0.0)	**	0 (0.0)	**	**0 (0.0)**	**
1–4	0 (0.0)	**	0 (0.0)	**	**0 (0.0)**	**
5–9	0 (0.0)	**	0 (0.0)	**	**0 (0.0)**	**
10–14	1 (<1.0)	**	0 (0.0)	**	**1 (<1.0)**	**
15–19	12 (4.5)	**	4 (21.1)	**	**16 (5.7)**	**
20–24	39 (14.8)	1.0	0 (0.0)	**	**39 (13.8)**	**0.5**
25–29	45 (17.0)	1.2	3 (15.8)	**	**48 (17.0)**	**0.7**
30–34	38 (14.4)	1.1	1 (5.3)	**	**39 (13.8)**	**0.6**
35–44	59 (22.3)	0.9	7 (36.8)	**	**66 (23.3)**	**0.5**
45–54	41 (15.5)	0.6	2 (10.5)	**	**43 (15.2)**	**0.3**
55–64	18 (6.8)	**	1 (5.3)	**	**19 (6.7)**	**
65–74	10 (3.8)	**	1 (5.3)	**	**11 (3.9)**	**
75–84	1 (<1.0)	**	0 (0.0)	**	**1 (<1.0)**	**
≥85	0 (0.0)	**	0 (0.0)	**	**0 (0.0)**	**
Unknown	0 (0.0)	**	0 (0.0)	**	**0 (0.0)**	**
**Total**	**264 (100)**	**0.5**	**19 (100)**	**	**283 (100)**	**0.3**
**Race/ethnicity**						
White, non-Hispanic	128 (48.5)	0.4	12 (63.2)	**	**140 (49.5)**	**0.2**
Black, non-Hispanic	79 (29.9)	1.0	4 (21.1)	**	**83 (29.3)**	**0.5**
American Indian/Alaska Native, non-Hispanic	9 (3.4)	**	0 (0.0)	**	**9 (3.2)**	**
Asian/Pacific Islander	2 (<1.0)	**	1 (5.3)	**	**3 (1.1)**	**
Hispanic^††^	45 (17.0)	0.8	2 (10.5)	**	**47 (16.6)**	**0.4**
Other	1 (<1.0)	**	0 (0.0)	**	**1 (<1.0)**	**
Unknown	0 (0.0)	**	0 (0.0)	**	**0 (0.0)**	**
**Total**	**264 (100)**	**0.5**	**19 (100)**	**	**283 (100)**	**0.3**

* Percentages might not total 100% due to rounding.

^†^ Per 100,000 population.

^§^ The term “legal intervention” does not denote the lawfulness or legality of the circumstances surrounding the death.

^¶^ Alaska, Colorado, Georgia, Kentucky, Maryland, Massachusetts, Michigan, North Carolina, New Jersey, New Mexico, Ohio, Oklahoma, Oregon, Rhode Island, South Carolina, Utah, Virginia, and Wisconsin.

** Rate is not reported when number of decedents is <20 or when race/ethnicity or age categories are “Other” or “Unknown.”

^††^ Includes persons of any race.

#### Method and Location of Injury

Firearms were used in almost all legal intervention deaths (94.7%) (Table 12). Legal intervention deaths occurred most frequently in a house or apartment (41.7%), followed by a street or highway (24.0%) and a motor vehicle (8.5%) (Table 12).

**TABLE 12:** Number, percentage,* and rate^†^ of legal intervention^§^ deaths, by decedent's sex, method used, and location in which injury occurred — National Violent Death Reporting System, 18 states,^¶^ 2014

	Male	Female	Total	
Characteristic	No. (%)	No. (%)	No. (%)	Rate
**Method**				
Firearm	251 (95.1)	17 (89.5)	**268 (94.7)**	**0.3**
Motor vehicle (e.g., car, bus, motorcycle, or other transport vehicle)	3 (1.1)	0 (0.0)	**3 (1.1)**	**
Sharp instrument	2 (<1.0)	0 (0.0)	**2 (<1.0)**	**
Drowning	1 (<1.0)	0 (0.0)	**1 (<1.0)**	**
Fall	1 (<1.0)	0 (0.0)	**1 (<1.0)**	**
Hanging/strangulation/suffocation	1 (<1.0)	0 (0.0)	**1 (<1.0)**	**
Personal weapons (e.g., hands, feet, fists)	1 (<1.0)	0 (0.0)	**1 (<1.0)**	**
Poisoning	1 (<1.0)	0 (0.0)	**1 (<1.0)**	**
Blunt instrument	0 (0.0)	0 (0.0)	**0 (0.0)**	**
Fire/burns	0 (0.0)	0 (0.0)	**0 (0.0)**	**
Intentional neglect	0 (0.0)	0 (0.0)	**0 (0.0)**	**
Other (single method)	3 (1.1)	1 (5.3)	**4 (1.4)**	**
Unknown	0 (0.0)	1 (5.3)	**1 (<1.0)**	**
**Total**	**264 (100)**	**19 (100)**	**283 (100)**	**0.3**
**Location**				
House, apartment	107 (40.5)	11 (57.9)	**118 (41.7)**	**0.1**
Street/highway	65 (24.6)	3 (15.8)	**68 (24.0)**	**0.1**
Motor vehicle	21 (8.0)	3 (15.8)	**24 (8.5)**	**0.0**
Parking lot/public garage/public transport	19 (7.2)	0 (0.0)	**19 (6.7)**	**
Commercial/retail area	17 (6.4)	0 (0.0)	**17 (6.0)**	**
Natural area	10 (3.8)	0 (0.0)	**10 (3.5)**	**
Office building	3 (1.1)	0 (0.0)	**3 (1.1)**	**
Park, playground, sports/athletic area	3 (1.1)	0 (0.0)	**3 (1.1)**	**
Farm	2 (<1.0)	0 (0.0)	**2 (<1.0)**	**
Industrial or construction area	1 (<1.0)	1 (5.3)	**2 (<1.0)**	**
Jail/prison	2 (<1.0)	0 (0.0)	**2 (<1.0)**	**
Supervised residential facility	2 (<1.0)	0 (0.0)	**2 (<1.0)**	**
Bar/nightclub	1 (<1.0)	0 (0.0)	**1 (<1.0)**	**
Hotel/motel	1 (<1.0)	0 (0.0)	**1 (<1.0)**	**
Abandoned house/building/warehouse	0 (0.0)	0 (0.0)	**0 (0.0)**	**
Hospital or medical facility	0 (0.0)	0 (0.0)	**0 (0.0)**	**
Preschool/school/college/school bus	0 (0.0)	0 (0.0)	**0 (0.0)**	**
Railroad tracks	0 (0.0)	0 (0.0)	**0 (0.0)**	**
Other	9 (3.4)	1 (5.3)	**10 (3.5)**	**
Unknown	1 (<1.0)	0 (0.0)	**1 (<1.0)**	**
**Total**	**264 (100)**	**19 (100)**	**283 (100)**	**0.3**

* Percentages might not total 100% due to rounding.

^†^ Per 100,000 population.

^§^ The term “legal intervention” does not denote the lawfulness or legality of the circumstances surrounding the death.

^¶^ Alaska, Colorado, Georgia, Kentucky, Maryland, Massachusetts, Michigan, North Carolina, New Jersey, New Mexico, Ohio, Oklahoma, Oregon, Rhode Island, South Carolina, Utah, Virginia, and Wisconsin.

** Rate is not reported when number of decedents is <20.

#### Precipitating Circumstances

Precipitating circumstances were identified for 99.6% of legal intervention deaths. Approximately 88.3% were reportedly precipitated by another crime (Table 13); in 65.9% of those, the crime was in progress at the time of the incident. The type of crime most frequently precipitating the legal intervention death was assault/homicide (65.5%), followed by robbery (9.6%), motor vehicle theft (6.4%), burglary (5.6%), and drug trade (2.0%). Substance abuse problems other than alcohol abuse (25.2%) and current diagnosed mental health problem (21.6%) were the most common circumstances related to mental health/substance abuse. Other notable precipitating circumstances were an argument or conflict (13.8%), being a perpetrator of interpersonal violence within the past month (8.9%), drug involvement (8.9%), and family relationship problems (6.4%). In 10.3% of legal intervention deaths with known circumstances, IPV was identified as a contributing factor (Table 13). The decedent reportedly used a weapon in 72.7% of legal intervention deaths (Table 13). A recent crisis (within the previous or upcoming 2 weeks) was cited in 13.8% of legal intervention deaths (Table 13).

**TABLE 13:** Number* and percentage^†^ of legal intervention^§^ deaths, by precipitating circumstances and decedent's sex — National Violent Death Reporting System, 18 states,^¶^ 2014

	Male	Female	Total
Precipitating circumstances	No. (%)	No. (%)	No. (%)
**Mental health/substance abuse**			
Other substance abuse problem (excludes alcohol)	67 (25.5)	4 (21.1)	**71 (25.2)**
Current diagnosed mental health problem	52 (19.8)	9 (47.4)	**61 (21.6)**
History of ever being treated for a mental health problem	47 (17.9)	6 (31.6)	**53 (18.8)**
Current mental health treatment	31 (11.8)	5 (26.3)	**36 (12.8)**
Alcohol problem	25 (9.5)	4 (21.1)	**29 (10.3)**
Current depressed mood	19 (7.2)	2 (10.5)	**21 (7.4)**
Other addiction (e.g., gambling, sexual)	0 (0.0)	0 (0.0)	**0 (0.0)**
**Interpersonal**			
Intimate partner violence-related	27 (10.3)	2 (10.5)	**29 (10.3)**
Perpetrator of interpersonal violence within past month	24 (9.1)	1 (5.3)	**25 (8.9)**
Family relationship problem	15 (5.7)	3 (15.8)	**18 (6.4)**
Other relationship problem (nonintimate)	3 (1.1)	3 (15.8)	**6 (2.1)**
Jealousy (lovers’ triangle)	3 (1.1)	0 (0.0)	**3 (1.1)**
Victim of interpersonal violence within past month	0 (0.0)	0 (0.0)	**0 (0.0)**
**Life stressor**			
Crisis within previous or upcoming two weeks	33 (12.5)	6 (31.6)	**39 (13.8)**
Argument or conflict	35 (13.3)	4 (21.1)	**39 (13.8)**
Physical fight (two persons, not a brawl)	14 (5.3)	2 (10.5)	**16 (5.7)**
History of child abuse/neglect	3 (1.1)	2 (10.5)	**5 (1.8)**
**Crime and criminal activity**			
Precipitated by another crime	236 (89.7)	13 (68.4)	**249 (88.3)**
Crime in progress**	158 (66.9)	6 (46.2)	**164 (65.9)**
Drug involvement	23 (8.7)	2 (10.5)	**25 (8.9)**
Gang-related	2 (<1.0)	0 (0.0)	**2 (<1.0)**
Terrorist attack	0 (0.0)	0 (0.0)	**0 (0.0)**
**Legal intervention event**			
Victim used a weapon	195 (74.1)	10 (52.6)	**205 (72.7)**
Brawl	4 (1.5)	0 (0.0)	**4 (1.4)**
Victim was a bystander	1 (<1.0)	1 (5.3)	**2 (<1.0)**
Mentally ill suspect	1 (<1.0)	0 (0.0)	**1 (<1.0)**
Prostitution	1 (<1.0)	0 (0.0)	**1 (<1.0)**
Victim was a police officer on duty	0 (0.0)	0 (0.0)	**0 (0.0)**
Victim was an intervener assisting a crime victim	0 (0.0)	0 (0.0)	**0 (0.0)**
Random violence	0 (0.0)	0 (0.0)	**0 (0.0)**
Stalking	0 (0.0)	0 (0.0)	**0 (0.0)**
**Total legal intervention deaths with precipitating circumstances**	**263 (100)**	**19 (100)**	**282 (100)**

* Includes deaths with one or more precipitating circumstances. Total numbers do not equal the sums of the columns because more than one circumstance could have been present per decedent. Circumstances were unknown for one male decedent.

^†^ Denominator includes only those deaths with one or more precipitating circumstances. The sum of percentages in columns exceed 100% because more than one circumstance could have been present per decedent.

^§^ The term “legal intervention” does not denote the lawfulness or legality of the circumstances surrounding the death.

^¶^ Alaska, Colorado, Georgia, Kentucky, Maryland, Massachusetts, Michigan, North Carolina, New Jersey, New Mexico, Ohio, Oklahoma, Oregon, Rhode Island, South Carolina, Utah, Virginia, and Wisconsin.

** Denominator includes only those decedents involved in an incident that was precipitated by another crime.

### Unintentional Firearm Deaths

#### Sex, Race/Ethnicity, and Age Group

The 18 NVDRS states included in this report collected data concerning 144 incidents involving 144 unintentional firearm deaths in 2014 (Table 14). A total of 69 (47.9%) of these unintentional fatal injuries were self-inflicted, and 62 (43.1%) were known to be inflicted by another person; for the remaining 13 (9.0%), it was unknown who inflicted the injury. Males accounted for 84.7% of decedents. The majority (67.4%) were non-Hispanic whites, followed by non-Hispanic blacks (25.0%). Persons aged 10–24 years accounted for approximately 40% of all unintentional firearm deaths (Table 14).

**TABLE 14:** Number* and percentage^†^ of unintentional firearm deaths, by decedent's sex, race/ethnicity, age group, location of injury, and type of firearm — National Violent Death Reporting System, 18 states,^§^ 2014

Characteristic	No. (%)
**Sex**	
Male	122 (84.7)
Female	22 (15.3)
**Total**	**144 (100)**
**Race/ethnicity**	
White, non-Hispanic	97 (67.4)
Black, non-Hispanic	36 (25.0)
American Indian/Alaskan Native	3 (2.1)
Asian/Pacific Islander	1 (<1.0)
Hispanic^¶^	7 (4.9)
**Total**	**144 (100)**
**Age group (yrs)**	
1–4	9 (6.3)
5–9	9 (6.3)
10–14	13 (9.0)
15–19	18 (12.5)
20–24	26 (18.1)
25–29	11 (7.6)
30–34	10 (6.9)
35–44	10 (6.9)
45–54	10 (6.9)
55–64	14 (9.7)
65–74	10 (6.9)
75–84	3 (2.1)
≥85	1 (<1.0)
**Total**	**144 (100)**
**Location**	
House, apartment	110 (76.4)
Natural area	7 (4.9)
Street/highway	6 (4.2)
Motor vehicle	5 (3.5)
Commercial/retail area	2 (1.4)
Farm	2 (1.4)
Hotel/motel	2 (1.4)
Bar/nightclub	1 (<1.0)
Park, playground, sports/athletic area	1 (<1.0)
Parking lot/public garage/public transport	1 (<1.0)
Other**	1 (<1.0)
Unknown	6 (4.2)
**Total**	**144 (100)**
**Firearm type**	
Handgun	86 (59.7)
Rifle	25 (17.4)
Shotgun	20 (13.9)
Other firearm	1 (<1.0)
Unknown	12 (8.3)
**Total**	**144 (100)**

* No. deaths = 144.

^†^ Percentages might not total 100% due to rounding.

^§^ Alaska, Colorado, Georgia, Kentucky, Maryland, Massachusetts, Michigan, North Carolina, New Jersey, New Mexico, Ohio, Oklahoma, Oregon, Rhode Island, South Carolina, Utah, Virginia, and Wisconsin.

^¶^ Includes persons of any race.

** Includes military training exercise, private land campsites, and private hunting land attached to homes.

#### Firearm Type and Location of Injury

Handguns were involved in 59.7% of unintentional firearm deaths, rifles in 17.4%, and shotguns in 13.9%. (Table 14). Of all unintentional firearm deaths, 76.4% occurred in a house or apartment, followed by natural areas (4.9%) and on the street/highway (4.2%) (Table 14).

#### Context of the Injury and Associated Circumstances

The context of the injury or associated circumstances were known for 91.7% of unintentional firearm deaths (Table 15). Overall, the most common context of injury was playing with a gun (40.9%), followed by cleaning the gun (12.9%) and showing the gun to others (9.8%). The most common associated circumstance was unintentionally pulling the trigger (26.5%), followed by mistakenly thinking the gun was unloaded (15.2%) and mistakenly thinking the magazine was disengaged (8.3%) (Table 15).

**TABLE 15:** Number* and percentage^†^ of unintentional firearm deaths, by context and circumstances of injury — National Violent Death Reporting System, 18 states,^§^ 2014

Characteristic	No. (%)
**Context of injury**	
Playing with gun	54 (40.9)
Cleaning gun	17 (12.9)
Showing gun to others	13 (9.8)
Hunting	11 (8.3)
Loading/unloading gun	9 (6.8)
Target shooting	4 (3.0)
Celebratory firing	0 (0.0)
Other context of injury	33 (25.0)
**Circumstances of injury**	
Unintentionally pulled trigger	35 (26.5)
Thought gun was unloaded	20 (15.2)
Thought unloaded, magazine disengaged	11 (8.3)
Gun fired due to defect or malfunction	5 (3.8)
Gun was mistaken for a toy	4 (3.0)
Thought gun safety was engaged	2 (1.5)
Bullet ricocheted	2 (1.5)
Gun fired while holstering	1 (<1.0)
Gun was dropped	1 (<1.0)
Gun fired while handling safety lock	1 (<1.0)
Other mechanism of injury	32 (24.2)
**Total unintentional firearm deaths with precipitating circumstances**	**132 (100)**

* Includes 132 deaths with one or more circumstances known. Circumstances were unknown for 12 deaths.

^† ^Percentages might exceed 100% because one or more circumstances could have been known per death.

^§^ Alaska, Colorado, Georgia, Kentucky, Maryland, Massachusetts, Michigan, North Carolina, New Jersey, New Mexico, Ohio, Oklahoma, Oregon, Rhode Island, South Carolina, Utah, Virginia, and Wisconsin.

### Deaths of Undetermined Intent

#### Sex, Race/Ethnicity and Age Group

The 18 NVDRS states included in this report collected data concerning 2,257 deaths in 2014 for which a determination of intent could not be made (crude rate: 2.1 per 100,000 population). Rates were higher among males than among females (2.7 and 1.6 per 100,000 population, respectively), and non-Hispanic whites accounted for 74.1% of deaths of undetermined intent. Non-Hispanic American Indian/Alaska Native decedents had the highest rate (3.0 per 100,000 population). Nearly half (46.3%) of persons for whom the manner of death was undetermined were aged 35–54 years. Rates were highest among adults aged 45–54 years (4.0 per 100,000 population).

#### Method of Injury

The most common method of injury in deaths of undetermined intent was poisoning (73.8%). No other method accounted for >4.0% overall. Opiates (including heroin and prescription pain medications) were the most frequently tested (75.7%) and most frequently detected (77.8% of those tested) substances among this group.

#### Precipitating Circumstances

Precipitating circumstances were known in approximately 86.6% of deaths of undetermined intent. The most common circumstances were physical health problems (18.9%), a crisis during the preceding or upcoming 2 weeks (16.5%), and intimate partner problems (8.4%). Nonalcohol substance abuse problems (65.7%) and alcohol problems (29.1%) were common, and 37.8% had a current diagnosed mental health problem. Among those with a current diagnosed mental health problem, depression/dysthymia (59.5%), anxiety disorder (23.5%), and bipolar disorder (23.1%) were the most common diagnoses. 

## Discussion

Violent deaths occur among persons of all ages, races, and ethnicities. NVDRS data can help identify populations particularly affected by violence. The system not only provides details on specific manners of violent deaths but also identifies cross-cutting risk factors for multiple types of violence. These details can increase knowledge about the circumstances associated with violence and can help public health authorities develop data-informed, effective approaches to violence prevention.

Violence is preventable. CDC has developed technical packages that present a collection of strategies that represent the best available evidence to help communities and states prevent or reduce public health problems like violence (*10*). Each technical package — child abuse and neglect, youth violence and associated risk factors, IPV, sexual violence, and suicide — identifies strategies and approaches that are representative of different levels of the social ecology intended to affect individual behaviors as well as the relationship, family, school, community, and societal factors that influence risk and protective factors for violence.

As NVDRS data demonstrate, the majority of violent deaths are suicides, making suicide prevention an important priority. CDC’s suicide technical package identifies seven primary strategies that have the greatest potential to prevent suicide and to reduce the immediate and long-term consequences of suicidal behavior (*11*). These strategies include strengthening economic supports, strengthening access and delivery of suicide care, creating protective environments, promoting connectedness, teaching coping and problem-solving skills, identifying and supporting people at risk, and lessening harms and preventing future risk. The specific approaches to advance these strategies, including strengthening household financial security, community engagement, and parenting skills and family relationships, are intended to work in combination and reinforce each other to effectively prevent suicide and also have cross-cutting impacts on other forms of violence (*11*).

NVDRS data underscore the importance of relationship problems as a risk factor for suicides and homicides and the importance of developing social and emotional skills (e.g., communication, conflict resolution, and empathy) and supportive relationships as protective factors (*12*). Violence is a leading cause of death among youth, and early intervention is critical to the prevention of youth violence. CDC’s youth violence technical package emphasizes the preventive effects of youth skill development programs as well as prevention approaches that address relationships and influence school and community environments (*12*).

The promotion of safe, stable, nurturing relationships and environments also is a key component of the prevention of child abuse and neglect (*13*). CDC’s child abuse and neglect technical package highlights several strategies that increase the likelihood that children will have safe, stable, nurturing relationships and environments. These strategies include strengthening economic support to families, changing social norms to support parents and positive parenting, providing quality care and education early in life, and enhancing parenting skills to promote healthy child development (*14*). Five percent of all homicides captured by NVDRS in 2014 (approximately 200 deaths) were related to abuse or neglect by a caretaker (the vast majority of which were among minors), approximately the same percentage as those related to gang violence and exceeding those related to many other homicide circumstances.

NVDRS homicide circumstance data also indicate that approximately half of homicides with female victims are related to IPV. Support for survivors of IPV to increase safety and lessen harms is an important part of prevention (*15*). The U.S. Preventive Services Task Force recommends screening women of childbearing age for IPV and referring women who screen positive to intervention services (*16*). Screening and counseling about abuse should be done in a culturally sensitive and supportive way to address concerns about health and safety (*17*). An intervention with multiple counseling sessions to assess the risk for danger, discuss prevention options, develop a safety plan, and share appropriate community resources has been reported to reduce recurrence of partner violence among pregnant women (*18*). Other prevention approaches focus on empowering bystanders in the prevention of IPV and sexual violence (*15*,*19*) and primary prevention through teaching young persons about safe and healthy relationship behaviors in the early dating years (*15*,*20*–*22*). These approaches and others (e.g., parenting skill and family relationship programs and engaging men and boys as allies in prevention) are described in CDC’s recently released IPV prevention technical package (*15*). Evidence exists for the effectiveness of these programs (*15*). For example, Safe Dates, which focuses on reducing dating violence among adolescents by enhancing awareness about abusive relationships, changing norms, and teaching skills to develop healthy relationships, has been reported to reduce long-term physical and sexual dating violence (*20*) and other types of youth violence (*21*). IPV prevention efforts can benefit from a comprehensive approach that also addresses community- and system-level factors by creating protective environments and strengthening economic supports for families (e.g., through organizational and workplace policies) (*15*).

Substance use also frequently precedes suicidal and interpersonal violent behavior (*23*). Poisoning is the most common suicide method for women, the third most common suicide method overall, and the leading mechanism of deaths of undetermined intent. Seventy-five percent of all decedents were tested, and approximately 88% of decedents with a history of substance abuse had one or more substances present in their system at the time of death. Opioids and over-the-counter drugs were the substances that most frequently caused death in isolation; opioids in combination with benzodiazepines or antidepressants were some of the more commonly occurring combinations of substances that caused death. Unintentional opioid overdose has been recognized as an epidemic (*24*), and these findings mirror concerns regarding the overdose potential of opioids. In response to these concerns, CDC recently issued an opioid prescribing guideline to encourage safer prescribing practices aimed at reducing the likelihood of patient abuse and overdose (*25*). In addition, taking into account that more decedents with a history of substance abuse than those without such a history have drugs in their system at the time of death, these findings suggest that comprehensive violence prevention efforts can benefit from strategies that address ongoing substance abuse problems.

NVDRS data can be used to define public health priorities, develop and evaluate programs and policies, and conduct research regarding violent deaths at the state level. For example, a recently published article using North Carolina Violent Death Reporting System (NCVDRS) data focused on improving the case ascertainment of pregnancy-associated suicides and homicides (*26*). By linking NCVDRS data to North Carolina maternal mortality data, 55.6% more pregnancy-associated (i.e., among women who were pregnant or postpartum) violent deaths were identified, resulting in higher mortality ratios for suicide (2.3 versus 3.3 deaths per 100,000 live births) and homicide (3.9 versus 6.2 deaths per 100,000 live births). Information provided by NCVDRS data indicated that 18% of suicides were related to postpartum depression and 65.5% of homicides were related to IPV. That analysis not only highlights the feasibility of linking NCVDRS with maternal mortality surveillance data but also underscores the importance of using multiple data systems to provide better estimates of the magnitude and circumstances related to pregnancy-associated violent deaths in North Carolina, which could guide prevention strategies. Data from state VDRS programs have been used more broadly to develop statewide violence prevention plans. For example, Wisconsin VDRS data were used to develop the statewide suicide prevention strategy (*27*).

NVDRS data also have been used in states to develop programs for veterans. For example, Colorado VDRS data are incorporated in general and topical reports concerning suicides among veterans. One report examined first responders and found that suicide victims who were first responders were more likely to have been veterans than the general population of suicide victims in their state (*28*). Findings from their data led to enhancing ManTherapy, an online suicide prevention program for men, to promote resources focused on positive mental and physical health for first responders, active military personnel, veterans, and their families (https://mantherapy.org).

At the national level, NVDRS data are relevant to two national prevention initiatives: the National Strategy for Suicide Prevention and Healthy People 2020 (*29*,*30*). The National Strategy for Suicide Prevention is a comprehensive national agenda for suicide prevention (*29*). Healthy People 2020 includes specific objectives for reducing the number of suicides, homicides, and firearm-related deaths and increasing the number of states that link data on violent deaths from death certificates, law enforcement reports, and coroner/medical examiner reports at state and local levels (*30*). Unlike other sources of data, NVDRS allows changing patterns in circumstances and risk profiles to be examined, which can affect how the rates are interpreted, help guide prevention activities, and monitor progress toward objectives.

Updating to an online platform has simplified NVDRS system operations and management, improved timeliness of data entry and reporting, and enhanced flexibility to adapt quickly to changing information needs in violence surveillance (*2*). Changes to the system have also improved its capacity for future expansion to additional states (*2*).

## Limitations

The findings in this report are subject to at least eight limitations. First, NVDRS data are available from a limited number of states and therefore are not nationally representative. Second, the availability, completeness, and timeliness of data are dependent on partnerships among state VDRS and state health departments, vital statistics registrars’ offices, coroners/medical examiners, and law enforcement personnel. Data sharing and communication among partners are particularly challenging when states have independent county coroner systems rather than a centralized coroner/medical examiner system, a large number of law enforcement jurisdictions, or both. NVDRS incident data might be limited or incomplete for areas in which these data-sharing relations are not fully developed. Third, toxicology data are not collected consistently across all states or for all alcohol and drug categories. Toxicology testing is not conducted for all decedents; therefore, the percentage of those with positive results for specific substances might be affected by selective testing patterns in coroner/medical examiner offices (*31*). Fourth, abstractors are limited to the data included in the investigative reports they receive. Reports might not fully reflect all information known about an incident, particularly for homicides and legal intervention deaths, when data are less readily available until after a full investigation and adjudication are completed. Fifth, a single death might be classified differently in different documents (e.g., unintentional in a law enforcement report, homicide in a coroner/medical examiner report, and undetermined on the death certificate). NVDRS abstractors reconcile these discrepancies using standard NVDRS case definitions and select a single manner of death on the basis of all source documents; the manner of death assigned must be consistent with the manner of death noted in at least one source document. Sixth, variations in coding might occur depending on the abstractor’s level of experience. For this reason, CDC provides abstractor training, and states conduct blinded reabstraction of cases to test consistency and identify training needs. Seventh, medical and mental health information (e.g., type of condition and whether the decedent was currently receiving treatment) are not often captured directly from medical records but from coroner/medical examiner reports and the decedent’s family members and friends. Therefore, the completeness and accuracy of this information are limited by the knowledge of the informant. Finally, protective factor data (i.e., characteristics or circumstances that reduce the risk for violent death) are not collected by NVDRS because of the nature of death certificates, coroner/medical examiner reports, and law enforcement reports, which typically contain only circumstances associated with risk factors.

## Conclusion

Public health surveillance is the foundation for public health practice. Surveillance is essential to monitoring the prevalence and incidence of violence-related fatal injuries, defining priorities, and directing programmatic and violence-prevention activities (*32*). Development and expansion of NVDRS are crucial to public health efforts at the federal, state, and local levels to reduce violence and the personal, familial, and societal consequences and costs. Further efforts are needed to increase the number of states participating in NVDRS to include all 50 states, U.S. territories, and the District of Columbia, with the ultimate goal of full national representation.

## Footnotes

* The name of the Institute of Medicine was changed to the National Academy of Medicine, effective July 1, 2015.

† To be included in NVDRS, deaths of undetermined intent must have some evidence of the possibility that the intent was purposeful, including use of a weapon or other evidence that force was used to inflict the injury. Most commonly, the coroner/medical examiner is unsure whether the death was a suicide or unintentional.

§ In 2014, Arizona, Connecticut, Hawaii, Iowa, Illinois, Indiana, Kansas, Maine, Minnesota, New Hampshire, New York, Pennsylvania, Vermont, and Washington joined the system. In 2016, Alabama, California, Delaware, Louisiana, Missouri, Nebraska, Nevada, Puerto Rico, Washington, D.C., and West Virginia joined the system for a total of 42 states and territories participating. Fourteen of these states did not report data until 2015 and the other 10 states and territories did not report data until 2017.

¶ Frequencies and rates of violent deaths included in this report will differ slightly from the frequencies and rates of violent deaths reported by NVDRS WISQARS, which excludes nonresident deaths that occur in participating states (i.e., occurrent deaths). NVDRS tracks both resident and occurrent violent deaths in the overall data set, and the numbers in this report reflect both.

** Drug trade is defined as the buying, selling, or passing of drugs in exchange for goods or money. Drug involvement includes drug use in addition to drug trade.
